# Maillard Reaction: Mechanism, Influencing Parameters, Advantages, Disadvantages, and Food Industrial Applications: A Review

**DOI:** 10.3390/foods14111881

**Published:** 2025-05-26

**Authors:** Leina El Hosry, Vanessa Elias, Vanessa Chamoun, Malda Halawi, Philippe Cayot, Anthony Nehme, Elias Bou-Maroun

**Affiliations:** 1 Department of Nursing and Health Sciences, Notre Dame University—Louaize, Zouk Mosbeh, Zouk Mikael P.O. Box 72, Lebanon; vjelias@ndu.edu.lb (V.E.); vmchamoun@ndu.edu.lb (V.C.); 2 Department of Chemistry, International College, Hamra Bliss Street, Beirut P.O. Box 113-5373, Lebanon; 3 Institut Agro, Université Bourgogne Europe, INRAE, UMR PAM, Procédés Alimentaires et Microbiologiques, F-21000 Dijon, France; philipe.cayot@agrosupdijon.fr; 4 School of Business, Antonine University—Mejdlaya, Zgharta P.O. Box 40016, Lebanon; anthony.nehme@ua.edu.lb

**Keywords:** S-Maillard reaction, non-enzymatic browning, vitamin C degradation, acrylamide, melanoidins, advanced glycation end-products, nutritional effect, food processing

## Abstract

The Maillard reaction is a complex chemical reaction that occurs between nucleophilic groups, such as thiolates or amino groups primarily from amino acids, peptides, proteins, and carbonyl groups, particularly from reducing sugars. The pH value of the medium is a key parameter controlling the kinetics of the Maillard reaction, as it influences the concentration of nucleophilic groups. Other specific conditions of reaction medium such as temperature, reaction time (or residence time in a process), and water activity also significantly influence the Maillard reaction. Understanding the impact of these parameters is essential for optimizing the Maillard reaction to enhance sensory attributes, nutritional qualities, and product stability during the storage and distribution of the final products. The Maillard reaction is responsible for the formation of desirable sensory qualities such as flavor, aroma, color, and texture in cooked and thermally processed foods, in addition to the improvement of nutritional value and shelf life of foods. In contrast, there are limitations in its industrial applications, as it can also generate harmful compounds such as acrylamide, N(6)-carboxymethyllysine, furans, and heterocyclic amines, as well as undesired changes in the nutritional value of the food. This review provides an overview of the Maillard reaction’s mechanism, influencing parameters, pros and cons, as well as some food industrial applications.

## 1. Introduction

The Maillard reaction is classified as a non-enzymatic browning reaction, in contrast to enzymatic browning, which involves the polymerization of phenolic compounds. This enzymatic polymerization is an oxidation reaction catalyzed by polyphenol oxidase in the presence of oxygen, resulting in the formation of brown pigments. Similarly, the Maillard reaction also produces brown colorants, but it occurs without the involvement of any enzyme catalysis. In 1912, Louis Camille Maillard, a French chemist, discovered a browning phenomenon when he heated a solution containing a sugar and an amino acid. This observation led to the naming of the Maillard reaction as a non-enzymatic browning reaction.

However, the Maillard reaction produces more than just color: the reactions between amino acids, peptides, or proteins and reducing sugars also generate a wide range of flavor and aroma compounds. Many factors influence the reaction, such as temperature, time, water activity, pH, the type of amino acid and reducing sugars, and vitamin C degradation [1,2,3]. The reaction has an impact on the quality of heated foods, and its nutritional value.

The Maillard reaction has both advantages and disadvantages in the food industry. On the one hand, it contributes to the pleasant flavor (through the production of volatile aroma compounds) and the appealing color (through the production of pigment molecules) of many food products, such as bread, coffee, and chocolate [2,4,5,6]. On the other hand, it can cause off-flavors, flavor loss, discoloration, and the loss of nutritional value of proteins [7,8]. In the food industry, flavor and color, whether desirable or undesirable, are critical in the production of products with consistent sensory quality. The Maillard reaction influences both the odor (perceived by the nose) and the taste (perceived by the tongue) of food. For example, during the roasting of cocoa beans, the Maillard reaction can modulate bitterness, thereby affecting the overall taste profile [9]. Contradictory information about the effects of Maillard reaction products (MRPs) on health suggests that more research is needed to expand the advanced glycation end-products and MRPs database, as well as to develop methods to reduce the formation of MRPs in home-cooked and processed food [6,10]. The Maillard reaction is also important in the brewing industry, where it influences the flavor and color of beer.

The nutritional effects of the Maillard reaction are complicated and vary with the food matrix and the duration of the reaction [11,12,13,14]. The reaction can lead to the formation of both beneficial and harmful substances. For instance, it can generate antioxidants such as melanoidins, which have been associated with potential health benefits. However, the same process may also result in the formation of harmful compounds like acrylamide, a substance that has been linked to cancer in animal studies [15]. Moreover, protein oxidation and the Maillard reaction are both involved in the molecular aging of proteins [16]. In fact, Maillard reactions are considered one of the contributing factors to the aging process in humans.

The purpose of this review is to present a comprehensive overview of the Maillard reaction’s mechanism and the various factors that influence it, including temperature, time, water activity, pH, the presence of amino acids and reducing sugars, and vitamin C degradation. Additionally, this review explores the advantages, disadvantages, and food industrial applications of the Maillard reaction.

## 2. The Maillard Reaction Mechanism

**Figure 1:**
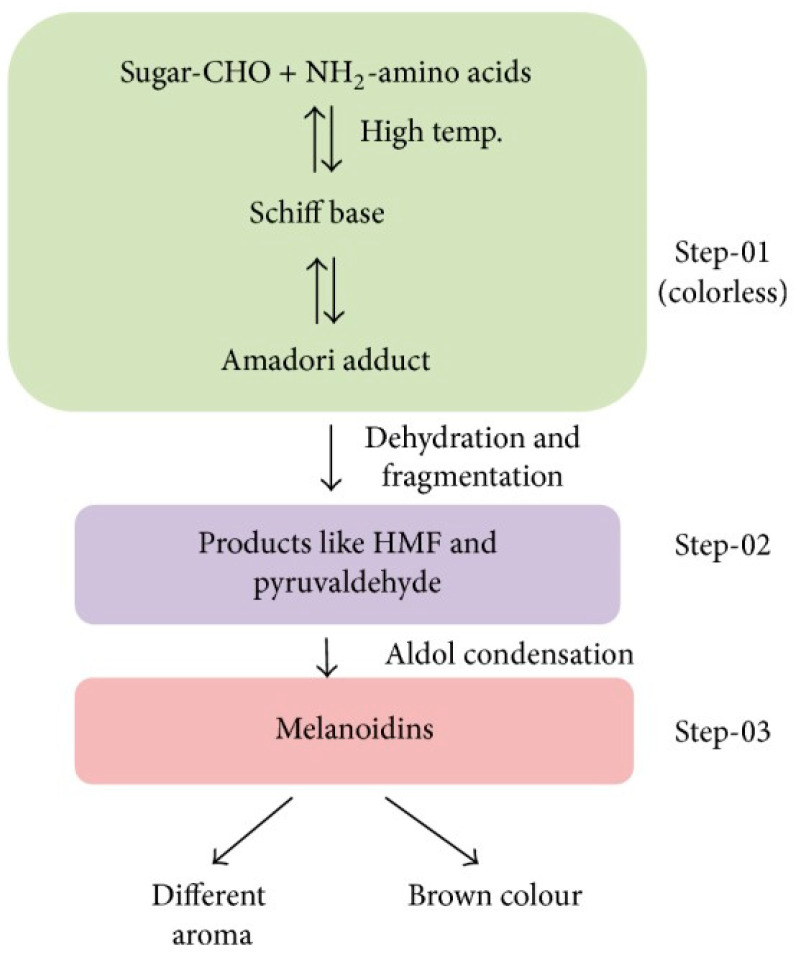
Diagram illustrating the Maillard reaction and the creation of flavors in food [4]. Step 1: Early stage. Step 2: Intermediate stage. Step 3: Advanced or final stage.

The Maillard reaction is a chemical reaction between amino acid residues and reducing sugars that leads to the formation of melanoidins, contributing to the distinct flavor and brown color of foods. This reaction initially forms glycosylamines, which subsequently rearrange into ketosamines and undergo further reactions to produce various flavor compounds and brown nitrogenous polymers, ultimately resulting in the characteristic browning of food (Figure 1) [1].

**Figure 2:**
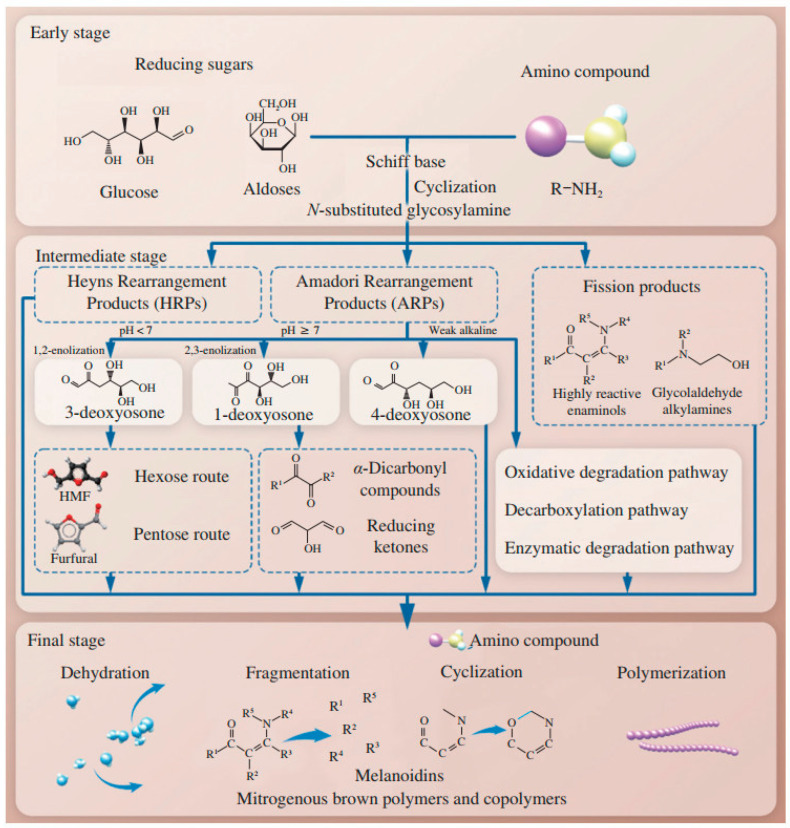
Representation of the three stages of the Maillard reaction [17]. The early stage is odorless and colorless; it corresponds to the condensation between a carbonyl group and an amino group. The intermediate stage gives rise to aroma compounds, mainly with heterocyclic structures. The final stage is responsible for the formation of melanoidins, which are brown polymers.

The Maillard reaction can be divided into roughly three stages (Figure 2).

### 2.1. Stage 1: Early Maillard Reaction

**Figure 3:**
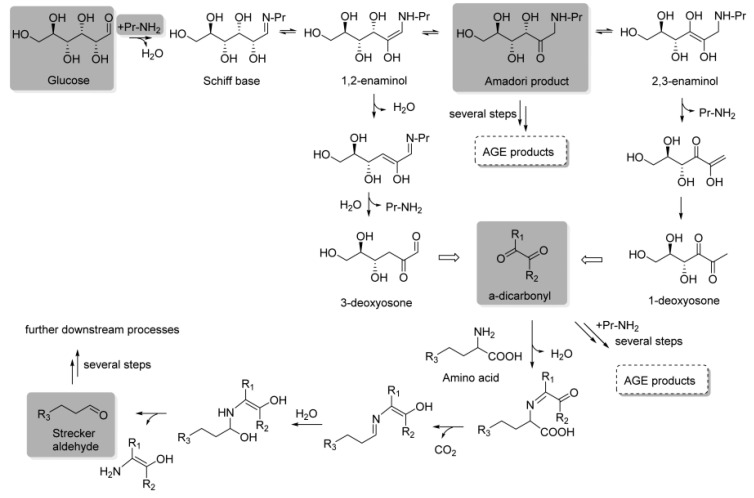
Simplified Maillard reaction between glucose, a reducing sugar, and an amine group of a protein to form a Schiff base, which can rearrange to an Amadori product; in the case of ketoses, it can also rearrange into a Heyns product via an 1,2-enaminol. Through enolization, the Amadori product leads to 1,2-enaminol or 2,3-enaminol and forms deoxyosones, α-dicarbonyl compounds. These can rapidly react rapidly with other nucleophiles to form Strecker aldehydes. Some of the Maillard reaction intermediates can generate AGEs (advanced glycation end-products) [2].

The first stage of the reaction, known as the early Maillard reaction, involves a series of reactions that includes the initial condensation by nucleophile addition, rearrangement, and fragmentation of reducing sugars and amino acids (Figure 2) [18]. The reaction begins with the condensation of the carbonyl group of the reducing sugar with the nucleophilic amino group of an amino acid to form a Schiff base or an Amadori rearrangement product (Figure 3) [19]. These initial products are reversible and can undergo further transformations. An important step in the early Maillard reaction mechanism is the Amadori rearrangement where a carbonyl group migrates from the C1 position of the reducing sugar to the C2 position, leading to the formation of a more stable ketoamine compound [20]. The fragmentation of the Amadori product is also significant, as it can undergo reactions such as degradation, dehydration, and rearrangement, leading to the formation of a mixture of reactive intermediates [21]. These intermediates can then undergo further transformation, leading to the formation of a wide range of MRPs, including flavor and aroma compounds, pigments, and other functional properties [22].

Factors like the nature and concentration of the reactants, reaction conditions such as temperature, pH, as well as the presence of catalysts and inhibitors, tend to influence the mechanism of the Maillard reaction. For instance, the nature of reducing sugar can influence the reaction rate and product formation [1,2]. Glucose and fructose have shown different reactivity towards amino-acid residue and therefore lead to the formation of distinct MRPs. Transition metal ions such as copper, iron, and zinc tend to accelerate the Maillard reaction by facilitating the formation of reactive intermediates. On the other hand, the presence of antioxidants, and reducing agents like ascorbic acid, can inhibit the Maillard reaction by scavenging free radicals and reducing the availability of reactive intermediates [23].

The Maillard reaction starts with a nucleophilic addition, where a Lewis base (acting as a nucleophile, Nü) reacts with carbonyl groups. This initial step is crucial in triggering the complex series of reactions that characterize the Maillard process. In foods, these nucleophiles typically include amino acid side-chain residues of proteins, such as guanidine groups ($H_{2}\ddot{N}-C\left(=\ddot{N}H\right)-\ddot{N}H-R$; Arg; pKa ≈ 12.5), thiolate ($R-{\ddot{S}}^{-}$; Cys; pKa ≈ 9), secondary amino groups ($R-H\ddot{N}-R^{\prime }$; His; pKa ≈ 6.5), and mainly primary amino groups ($R-\ddot{N}H_{2}$; Lys, pKa ≈ 10). A simple rule: the higher the pKa value, the stronger the base. However, when the pKa value is high, the reaction medium must have a correspondingly higher pH to increase the proportion of the nucleophilic base form (Nü) relative to its non-reactive conjugate acid form (Nu^+^H), as described by the equilibrium: Nu^+^H ⇌ H^+^ + Nü.

The carbonyl groups present in foods include aldehydes such as sugars (e.g., glucose, lactose, maltose) and aroma compounds (e.g., hexanal, vanillin), as well as ketones including sugars (e.g., fructose), vitamins (e.g., ascorbic acid), and aroma compounds (e.g., 2-nonanone). Carbonyl compounds can also originate from secondary oxidation products, including volatile aldehydes and ketones [24,25]. The Maillard reaction that follows lipid oxidation is one of the chemical pathways responsible for protein damage [26].

**Figure 4:**
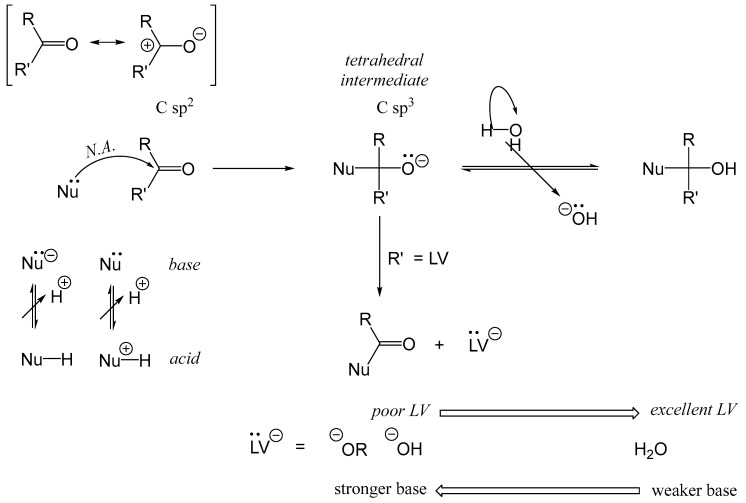
Early stage of Maillard reaction: nucleophilic addition. Addition of a nucleophile (Nü), a Lewis base, on a carbonyl, RR’C=O. LV: leaving group. In Maillard reaction, water (H_2_O) is the leaving group.

The nucleophilic addition reaction, which represents the initial step of the Maillard reaction, is depicted in Figure 4. In this reaction, a Lewis base (nucleophile) adds to an electrophilic carbonyl carbon, generating a tetrahedral intermediate. This intermediate serves as the starting point for various subsequent reaction pathways. When the nucleophile involved is a thiolate group (e.g., cysteinyl residues in proteins or cysteine as an amino acid), the reaction with carbonyl compounds is referred to as the S-Maillard reaction. Conversely, when the nucleophile is an amino group (e.g., lysyl residues, the terminal α-amino group of proteins or peptides, or lysine as an amino acid), the process is termed simply the Maillard reaction. Reactions involving arginine (Arg) or histidine (His) residues with carbonyl groups have been rarely described.

### 2.2. Stage 2: Intermediate Maillard Reaction with Lys Residues

During the intermediate stage of the Maillard reaction, various reactive intermediates are formed including α-dicarbonyl compounds, such as 1,2-dicarbonyls and 1,3-dicarbonyls, as well as compounds such as acrylamide, furans, and pyrazines (Figure 3) [27]. These reactive intermediates can undergo condensation, polymerization, cyclization, and rearrangement, which lead to the formation of a wide range of MRPs [17,22]. An important step at this stage is known as the Strecker degradation, which involves the degradation of amino acids through the reaction with α-dicarbonyl compounds (Figure 3) [17]. This results in the formation of volatile compounds known as Strecker aldehydes that contribute to the characteristic aroma and flavor of MRPs. Another significant reaction step involves the formation of furans, which are volatile compounds that play an important role regarding the nutty, caramel-like aroma of MRPs. They are formed from the dehydration and cyclization of sugars and amino acids, followed by further reactions with reactive intermediates [28].

**Figure 5:**
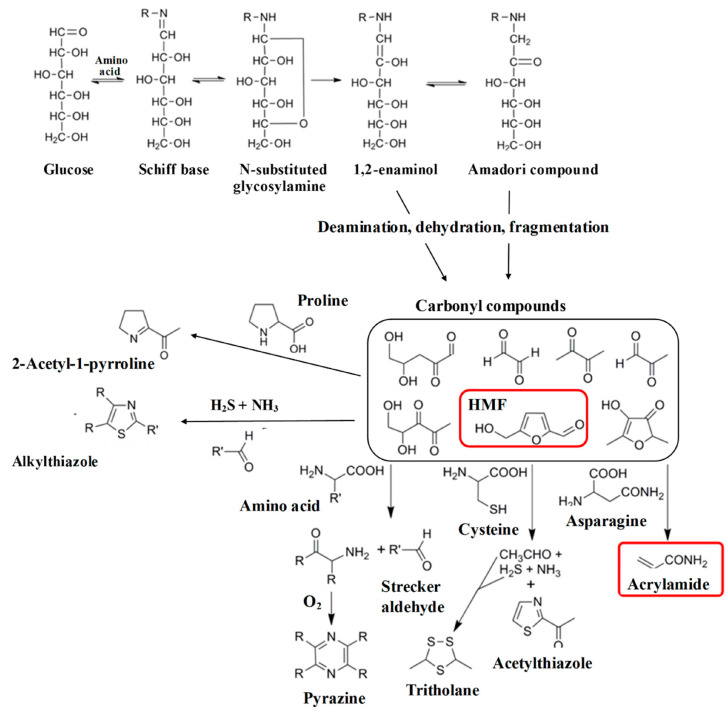
A simplified illustration of the Maillard reaction showing the formation of acrylamide, HMF in red, and other byproducts, many of which contribute to the taste and scent of fried, baked, roasted, and toasted foods. The subsequent deamination, dehydration, and fragmentation of these compounds generate highly reactive carbonyl compounds, including HMF. These can further interact with free amino acids, resulting in a diverse array of products [30].

During the intermediate Maillard reaction mechanism, acrylamide is formed through the reaction between asparagine, an amino acid found in many foods, and reducing sugars at high temperatures (Figure 5) [18]. The reaction continues with the formation of an intermediate, 3-aminopropionamide, which undergoes further intramolecular cyclization and dehydration to form acrylamide [29]. Additional reactions occur during the intermediate stage, including the formation of pyrazines that are responsible for the roasted, nutty flavors in several foods and beverages [22]. Pyrazines are formed through the reaction between amino-acid residues and carbohydrates, which result in the formation of intermediate products that undergo further reactions to form different pyrazines [22]. In addition, Figure 5 shows the formation of Hydroxymethylfurfural (HMF), a furanic compound containing aldehyde and alcohol functional groups. Like acrylamide, HMF is generated through the Maillard reaction during heat processing. Its formation involves an intermediate compound, 3-deoxyosone, that undergoes dehydration and cyclization to yield HMF [30].

### 2.3. Stage 3: Advanced Maillard Reaction

During the advanced stage of the Maillard reaction, a wide range of complex products are formed, ranging from low molecular weight compounds such as amino acids, sugars, and peptides to high molecular weight macromolecules such as melanoidins. These products are responsible for the characteristic colors, flavors, and textures of MRPs (Figure 1) [17]. An important reaction step at this stage is the formation of melanoidins, which are high molecular weight, brown-colored compounds with a complex structure. Melanoidins contribute to the characteristic color, flavor, and texture of MRPs, and they are also believed to possess health benefits due to their antioxidant and antimicrobial properties [31,32]. The advanced Maillard reaction mechanism is influenced by several factors that can affect reaction rate, pathway, and product formation, such as pH, temperature, reaction time, and the presence of catalysts or inhibitors. For example, during the presence of high pH or long reaction times, complex MRPs such as melanoidins can be formed [2].

**Figure 6:**
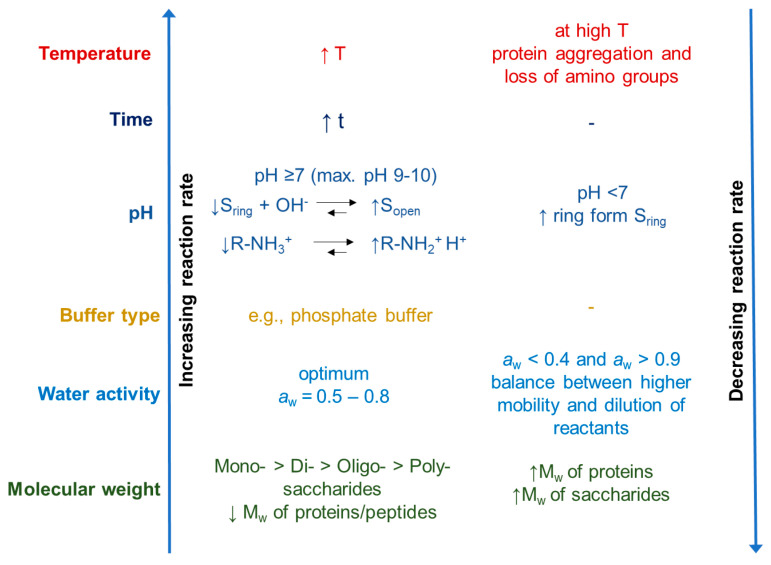
Key factors influencing the rate of the Maillard reaction. The reaction rate is modulated by several parameters, including temperature, time, pH, buffer type, water activity (a_w_), and molecular weight of reactants. Reaction acceleration is generally observed with increasing temperature and time, alkaline pH (≥7), moderate water activity (0.5–0.8), and the use of phosphate buffers. Low molecular weight carbohydrates and peptides also favor faster reaction kinetics. Conversely, reaction rate decreases under acidic conditions (pH < 7), extreme water activity values (a_w_ < 0.4 or >0.9), and with high molecular weight proteins or polysaccharides. At elevated temperatures, protein aggregation and amino group loss can inhibit reaction progress [34].

Many parameters influence the reaction rate of the Maillard reaction. Figure 6 is a schematic overview of the various environmental factors that may impact the rate of the Maillard reaction. In fact, the reactivity of proteins and carbohydrates decreases as molecular weight increases, primarily due to increased steric hindrance. Monosaccharides exhibit higher reactivity than di- or oligosaccharides when heated with whey proteins under similar conditions. Martinez-Alvarenga et al. [33] suggest that glycation is influenced by the preparation conditions, with temperature being the most important influence, followed by relative humidity and time, while the molar ratio of reactants has the least impact.

## 3. Influence of Different Parameters on the Maillard Reaction

### 3.1. Effect of pH on the Maillard Reaction

The pH value has a major impact on the kinetics of the Maillard reaction, as only the basic forms of functional groups—guanidino groups (H_2_N–C(=NH)–NH–R), secondary amino groups (R–NH–R’), primary amino groups (R–NH_2_), and thiolate groups (R–S^−^)—act as nucleophiles (Lewis bases, Nü) (Figure 1). The lower the pH, the lower the proportion of nucleophiles available, and thus the slower the reaction rate. The pKa value of sulfhydryl/thiolate groups in proteins is typically between 8 and 9 (R–SH ⇌ H^+^ + R–S^−^), while the pKa value for ammonium/amino groups is generally around 10 (R–NH_3_^+^ ⇌ H^+^ + R–NH_2_). At pH values below 6, the rate of the Maillard reaction is very low.

Variations in pH not only influence the rate and extent of the Maillard reaction but also affect the formation of flavor compounds and browning in foods. A slightly acidic environment slows the Maillard reaction but can lead to more pronounced browning and flavor development [2]. Extreme pH conditions can modify the reaction pathways, altering the final taste and color of cooked foods.

At higher pH levels, arginine residues (pKa ≈ 12; where the protonated guanidinium form, H_2_N–C(=N^+^H_2_)–NH–R, is in equilibrium with the nucleophilic base form, H_2_N–C(=NH)–NH–R) can also participate in the Maillard reaction [35,36,37]. Alkylation of arginyl residues produces different volatile compounds compared to those generated from alkylated lysyl residues, notably leading to the formation of pyrroles and pyrazines [38,39,40]. Furthermore, combined Maillard reactions involving both lysyl and arginyl residues can result in the formation of crosslinking products such as pentosidine [41].

The pH plays a crucial role in the Maillard reaction, as it affects the reaction rate, product properties, and safety. The Maillard reaction is a complex reaction that involves amino acids and reducing sugars, and the pH of the reaction environment influences the reactivity of these reactants. The protonation state of amino acids at different pH values affects their reactivity and extent of reaction with reducing sugars [34]. The pH can also influence the formation of reaction intermediates and products, as it can either promote or inhibit reaction pathways. Therefore, controlling the pH is essential in optimizing the Maillard reaction and controlling food quality [1].

The control of pH in the Maillard reaction is crucial for obtaining desirable sensory and quality attributes in food products. Food scientists and manufacturers manipulate pH values through ingredient selection and processing conditions to optimize the reaction and achieve the desired outcomes. For instance, modifying the pH level in food formulations can be achieved by adding acids or bases to adjust the pH. Controlling pH can help in enhancing or suppressing specific reactions to influence product appearance, taste, and texture. Additionally, pH control during the Maillard reaction is significant for the development of color and flavor in food products, such as baked goods, roasted coffee, and grilled meat [2]. Generally, pH equal to or under 5 is sufficient to stop the Maillard reaction in a moderate heating process (T ≤ 100 °C, time less than 24 h) or with a electro-assisted glycation [42].

#### 3.1.1. Effect of pH on the Reaction Rate and Product Formation

**Figure 7:**
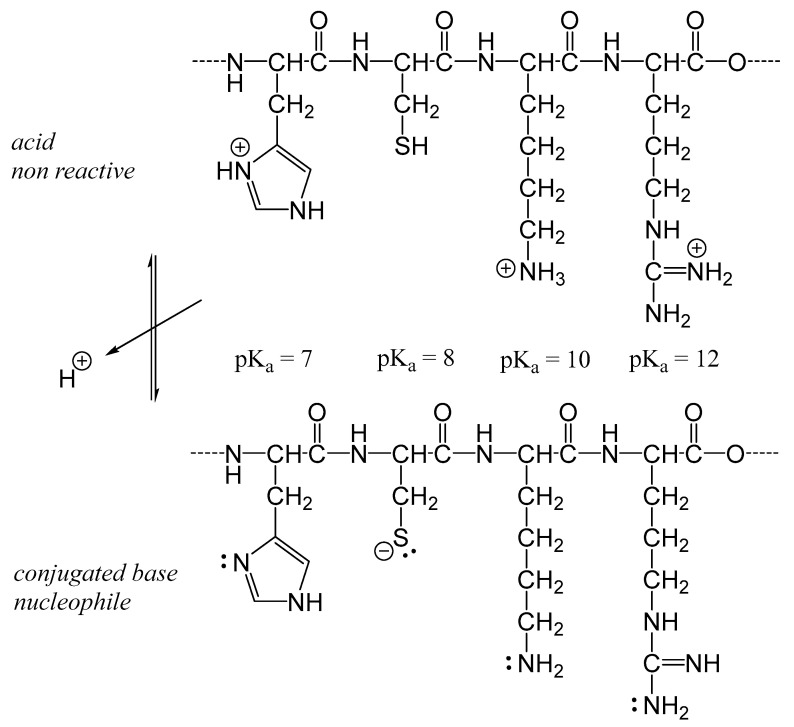
The base form of lateral chain of amino acid residues when the pH is closed (pH > pKa −3), equal or higher to pKa of His, Cys, Lys, Arg residues (left to right).

pH influences the rate of the Maillard reaction by altering the reactivity of the reactants and the generation of intermediate reactive species. In general, the Maillard reaction proceeds faster under basic conditions than at acidic pH levels. At acidic pH, amino groups are protonated, reducing their nucleophilicity and thereby limiting their reactivity (Figure 7). The increased proton concentration under acidic conditions also promotes the degradation of intermediate products, further hindering the progression of the Maillard reaction [43].

In contrast, alkaline conditions facilitate the Maillard reaction by promoting the formation of reactive carbonyl species. pH also significantly influences the types and quantities of MRPs. At low pH, fewer melanoidins—the dark brown pigments responsible for the characteristic color of MRPs—are formed [34]. This reduction in melanoidin production is primarily due to the decreased availability of reactive amino groups under acidic conditions. Nonetheless, under acidic conditions, the Maillard reaction can still promote the formation of furans, pyrazines, and other flavor compounds that contribute to desirable sensory properties [44].

In contrast, at higher pH values, greater amounts of melanoidin pigments are typically produced, resulting in a deeper brown coloration. Basic conditions enhance the formation of MRPs primarily through more efficient reactions between amino acids and reducing sugars, leading to the development of characteristic roasted and nutty flavors and aromas [45].

#### 3.1.2. Impact of pH on Product Safety and Stability

MRPs can have both beneficial and detrimental impacts on human health. Their negative influence is primarily due to the formation of advanced glycation end-products (AGEs), which have been linked to several chronic diseases, including diabetes, cardiovascular diseases, and Alzheimer’s disease [10]. The formation of AGEs depends on reaction conditions such as temperature, pH, and reactant concentrations.

The pH of the reaction medium significantly affects AGE production. Under acidic conditions, the protonation state of amino acids enhances their reactivity with carbonyl compounds, leading to increased AGE formation. In contrast, at alkaline pH levels, the generation of AGEs is often reduced, partly due to the formation of enolate intermediates, which are less likely to participate in the Maillard reaction to produce AGEs.

The stability of MRPs is also influenced by multiple factors, including pH, temperature, and humidity. Among these, pH plays a crucial role. At high pH values, MRPs can undergo hydrolysis or oxidation, leading to the formation of harmful metabolites [10]. Additionally, pH affects the types of volatile organic compounds (VOCs) generated during the Maillard reaction. It has been observed that pH has a greater impact on the formation of pyrazine derivatives than on pyrrole and pyridine derivatives. An alkaline environment particularly favors the production of nitrogen-containing heterocyclic VOCs [46].

### 3.2. Effect of Temperature on Maillard Reaction’s Rate

The kinetics of the Maillard reaction can be simplified according to Equation (1):

v = k · [R′R″C=O] · [R–NH_2_] (1)

or

v = k · [R′R″C=O] · [R–S^−^] (2)

where v is the rate of the reaction and k is the rate constant, [R′R″C=O] the concentration of reducing sugar, and [R–NH_2_] and [R–S^−^] are the concentrations of amino group and thiolate group respectively.

The rate constant k depends on the activation energy of the reaction and the temperature, according to the Arrhenius approximation or the Eyring–Polanyi equation. The higher the temperature, the faster the reaction. The activation energy (Ea) of the Maillard reaction depends on the substrates and the medium conditions. For example, Ea = 109 kJ⋅mol^−1^ for an aqueous solution of glucose and glycine (amino acid) at pH 5.5; Ea = 145 kJ⋅mol^−1^ for an aqueous solution of glucose and phenylalanine (amino acid) at pH 7; and Ea = 125 kJ⋅mol^−1^ for a powdered mixture of casein (protein, mainly lysyl residues) and lactose at water activity (a_w_) 0.52 and pH 6.5 [47].

A common misconception is that the Maillard reaction takes place exclusively at high temperatures. In reality, this reaction can also occur at much lower temperatures and, in some cases, even below freezing. Although the reaction rate significantly increases with heat, Maillard chemistry is not limited to thermal processing and can take place slowly under cold storage conditions. For instance, at 4 °C over a period of 12 months, the furosine content—a recognized marker of Maillard reaction progression—increased in stored royal jelly [48]. Similarly, changes in color (measured by absorbance at 420 nm) in frozen meat during storage at −4 °C and −20 °C indicate that Maillard reactions occur between ribose and lysyl groups in proteins [49,50]. Furthermore, assays of advanced glycation end-products (AGEs) in meatballs during frozen storage confirmed Maillard reaction activity at −18 °C over storage periods ranging from 30 to 120 days. The concentrations of ε-N-(carboxymethyl)lysine and ε-N-(carboxyethyl)lysine, measured by LC-MS/MS after acid hydrolysis, increased significantly during storage [51].

At high temperatures, typically above 120 °C, acrylamide can form as a byproduct of the Maillard reaction; it is classified as a potentially harmful compound and a probable human carcinogen by the International Agency for Research on Cancer (IARC). The Maillard reaction, influenced by factors such as food composition, temperature, and cooking time, leads to the generation of a diverse array of flavor compounds that contribute to the characteristic taste and aroma of cooked foods [29].

Temperature affects the reaction rate, which increases with the increasing temperature until a maximum rate is reached. The peak temperature is the temperature at which the Maillard reaction occurs most rapidly, and it varies depending on the food matrix, the types of reactants, and the specific reaction conditions [3]. High temperatures can cause undesirable reactions and the formation of undesirable products, including acrylamide and other harmful compounds. Therefore, it is essential to control the reaction’s temperature carefully to maximize the benefits of the Maillard reaction while minimizing the potential risks [30].

A study conducted by Zhou et al. [52] investigated the effect of temperature on the Maillard reaction between glucose and glycine. The mentioned researchers found that the maximum reaction rate occurred at 120 °C, and it decreased at temperatures above 120 °C or below 100 °C. The results showed that the Maillard reaction rate was affected by the temperature and that the optimum temperature for the reaction was 120 °C. Another study by Cao et al. [53] investigated the effect of temperature on the Maillard reaction between glucose and lysine. The researchers found that the Maillard reaction rate increased rapidly with increasing temperature from 100 °C to 120 °C and then became slower with higher temperatures. The results suggested that the rate of the Maillard reaction was influenced by the reaction temperature and that optimum reaction conditions for lysine-glycation of proteins were achieved at 110 °C.

#### 3.2.1. Effect of Temperature on Flavor and Color Development

Temperature plays a significant role in the development of flavor in the Maillard reaction: at lower temperatures, the Maillard reaction is relatively slow, and the formation of volatile flavor compounds is lower; at higher temperatures, the reaction rate increases, leading to the production of a higher number of volatile flavor compounds [54]. The thermal degradation of amino acids and intermediates due to high temperature is responsible for the formation of a wide range of aroma compounds in the Maillard reaction. Different types of flavor compounds are formed depending on the amino acid and sugar composition, reaction temperature, and reaction time [4,55]. For instance, the Maillard reaction between asparagine and glucose produces a relatively low amount of volatile flavor compounds at lower temperatures, while at higher temperatures above 140 °C, a high level of pyrazine and thiazole compounds like 2,5-dimethylpyrazine and 2-acetyl-2-thiazoline is observed. These compounds give a nutty and popcorn-like flavor to the food products [55]. Temperature also has a significant impact on the development of color during the Maillard reaction. At lower temperatures, the reaction is slow, and the browning of food products is relatively low. At higher temperatures, the reaction rate increases, leading to the production of more browning pigments such as melanoidins [54]. Melanoidins are responsible for the development of a brown color and a wide range of color shades during the Maillard reaction. The type and amount of reaction products depend on various factors, including the reaction temperature, type of sugar and amino acid, pH, and water activity [45].

#### 3.2.2. Effect of Temperature on the Food Product Safety

The Maillard reaction can lead to the formation of various potentially harmful compounds, including acrylamide, furan, and heterocyclic amines, which can be formed under specific reaction conditions [4,46,56]. Acrylamide is a known carcinogenic compound that can be formed during the cooking of foods at high temperatures, particularly in carbohydrate-rich foods like potato chips and French fries [57]. Similarly, furan is a toxic compound that can be formed during the thermal processing of foods like coffee and canned foods through the degradation of carbohydrates and amino acids during the Maillard reaction [58]. Heterocyclic amines are another group of potentially carcinogenic compounds that can be formed when meat and fish are cooked at high temperatures and are formed through the reaction between amino acids and creatinine and require temperatures above 150 °C [59]. The formation of these harmful compounds is temperature-dependent, meaning that higher temperatures increase the likelihood of their presence. Higher temperatures would favor the formation of pyrrole and pyridine derivatives, while the effect on pyrazine derivatives was less pronounced. Some pyrrole derivatives were only detected at a reaction temperature of 210 °C [46]. To minimize the formation of these potentially harmful compounds, it is essential to optimize the temperature conditions during the Maillard reaction. The selection of the optimal temperature conditions for product safety can be challenging as it involves balancing many factors, such as the desired sensory attributes, the type of food being processed, and the safety considerations [30]. However, for many food products, it is possible to choose temperatures that both allow for the desired sensory character and minimize the formation of harmful compounds. For instance, in the processing of coffee, roasting at lower temperatures can lead to the optimal flavor and aroma while minimizing the formation of furan. In the production of potato chips, the use of lower cooking temperatures can reduce the level of acrylamide without negatively affecting the desired sensory attributes [60].

### 3.3. Effect of Cooking Time

The relationship between time and the Maillard reaction is important in food preparation, as the duration of the reaction significantly impacts the development of flavors and aromas in cooked food. For instance, a short reaction time, such as 60 min, may result in the Maillard reaction being in its initial stages, leading to weaker meaty flavors and bitterness. As time progresses, the Maillard reaction intensifies, creating a diverse array of flavor compounds influenced by factors like food composition, cooking time, and the presence of air [4].

#### 3.3.1. Reaction Rate as a Function of Time

The rate of reaction in the Maillard reaction is often monitored as a function of time to determine the reaction progress and optimize processing conditions and can be measured in various ways, such as monitoring the consumption of reactants or the production of reaction products [2,13]. The progress of the Maillard reaction can be described by a reaction curve that displays the reaction rate as a function of time. These reaction curves can provide information about the optimal processing conditions for the Maillard reaction proceed effectively without affecting the product’s safety or quality [61]. Generally, the initial stage of the Maillard reaction is characterized by a slow reaction rate, followed by an acceleration phase where the reaction rate rapidly increases, and a final stage where the reaction rate slows down and eventually reaches a plateau. Monitoring the rate of reaction in the Maillard reaction can have various practical applications [34]. An example would be in the production of baked goods like bread; the rate of the Maillard reaction during baking can be monitored to optimize processing conditions like baking time and temperature. Similarly, in the production of roasted coffee, the Maillard reaction has a significant effect on the flavor and aroma of the coffee, and controlling the reaction rate during roasting can lead to the desired sensory attributes [62].

#### 3.3.2. Changes in Flavor and Color over Time

The MRPs flavor profile can change over time if the reaction continues to proceed beyond the desired state. The sensory changes can be attributed to the formation of advanced MRPs (AMPs) or oxidation of reaction products. AMPs result from the reaction between the MRPs in an autocatalytic process. These products can lead to undesirable sensory attributes such as bitterness and astringency [4]. Additionally, the products of the Maillard reaction can interact with oxygen molecules to form oxidative products that can contribute to off-flavors such as rancidity and stale flavors. The MRPs’ color can also change over time if the reaction continues beyond the desired point, leading to the formation of undesired pigments which are often characterized by the color shift from brown to black [63]. One group of undesired pigments formed during the Maillard reaction are melanoidins, and if they are present in large amounts in the food products, it can lead to the darkening of food products, ultimately causing undesirable sensory attributes [45].

### 3.4. Effect of Water Activity

**Figure 8:**
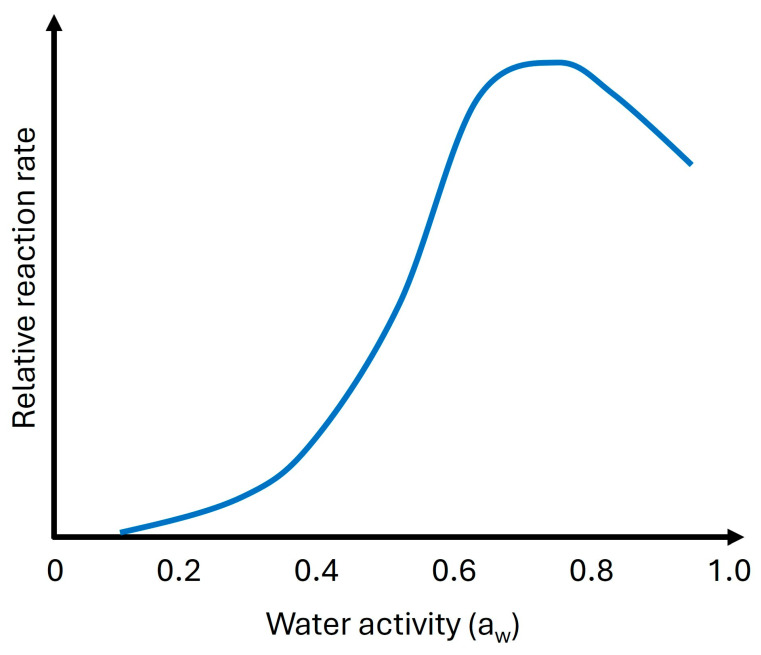
The impact of water activity on the rate of deteriorative reactions that occur in food systems [65].

The relationship between water activity and the Maillard reaction is significant in food processing. Water activity influences the rate and color of the Maillard reaction, with a peak reaction rate typically occurring around a water activity of 0.6 to 0.7 (Figure 8). As water activity approaches 0.70, the Maillard reaction and sugar browning accelerate, but beyond this point, the reaction slows due to excessive free water diluting the reactants [64].

#### 3.4.1. Importance of Water Activity

Water activity (a_w_) is an essential parameter in the Maillard reaction, as it affects the reaction rate, product properties, and safety. Water activity measures the amount of water available for chemical reactions, and it influences the availability of reactants and the mobility of molecules in the reaction system. The Maillard reaction is sensitive to water activity since the reaction requires enough water to facilitate the reaction of amino acids and reducing sugars. Low water activity can lead to decreased reaction rates or incomplete reaction, leading to the formation of unwanted intermediates as well as limiting the movement of the reactants, making it more challenging for them to come into contact and react. This can result in undesired sensory attributes such as a burnt or bitter taste [34].

#### 3.4.2. Influence of Water Activity on the Reaction Rate

Water activity influences the Maillard reaction rate by affecting the availability of reactants and reaction intermediates. The reaction rate is usually faster at higher water activity since water facilitates the movement and interaction of reactants, resulting in a higher collision rate between amino acids and reducing sugars. However, high water activity can also lead to the formation of unwanted products, decreased product stability, and decreased shelf life [5]. The Maillard reaction rate is typically highest in a limited water system compared to a high-water activity system and is due to fewer available water molecules that limit the reaction rate by inhibiting the transfer of amino acids and reducing sugars. Low water activity can also decrease the Maillard reaction rate since it limits the availability of water for chemical reactions [65].

#### 3.4.3. Effect of Water Activity on Product Texture and Quality

A low water activity level results in a hard, dense, and dry product because there is no free water for reactions or moisture redistribution, resulting in a loss of domain interconnectivity and porosity. In contrast, greater water activity results in a softer and moister product due to higher water mobility. Thus, determining the correct range of water activity is critical in generating the desirable textures of MRPs [66] and optimal overall quality.

The ideal water activity range allows the Maillard reaction to take place without causing any negative impacts on the end-product’s quality. Water activity values have a significant impact on the color development, scent, taste, and texture of the finished product [67]. Furthermore, water activity influences the Maillard reaction’s ultimate product color, with low water activity levels resulting in a darker product due to higher molecular crowding and more effective production of intermediate products, which leads to the formation of undesired brown melanoidins. Medium to high water activity levels result in light to dark brown products with the necessary balanced browning hue [68].

### 3.5. Presence of Amino Acids and Reducing Sugars

Amino acids and reducing sugars are the main reactants in the Maillard reaction, leading to the creation of diverse flavor compounds. Reducing sugars like glucose and ribose readily react with amino residues in proteins and free amino acids, initiating this complex chemical process [69].

Amino acids contribute to the flavor and nutritional quality of foods, while reducing sugars act as a source of energy and contribute to the sweetness of foods [13].

Amino acids are the primary building blocks of proteins, and their presence is required for the Maillard reaction to proceed [70]. During the Maillard reaction, amino acids undergo several chemical modifications, including deamination, decarboxylation, and cyclization, which result in the creation of a variety of reactive intermediates [71]. These reactive intermediates can subsequently undergo further reactions, resulting in the creation of a diverse spectrum of MRPs, such as flavor and fragrance molecules [70]. Different amino acids have varying degrees of reactivity with reducing sugars. For example, the amino acids lysine and arginine are extremely reactive and are frequently employed in Maillard reaction research. Different amino acids exhibit varying degrees of reactivity with reducing sugars and vice versa. For example, fructose interacts more easily with amino acids than glucose. The reactivity of reducing sugars towards amino acids is determined by a number of parameters, including the reducing sugar’s chemical structure, reactant concentration, and reaction circumstances such as temperature and pH [69].

#### 3.5.1. Influence of the Type and Concentration of Amino Acids and Reducing Sugars on the Reaction Rate and Product Formation

The type and concentration of amino acids significantly affect the rate and product formation of the Maillard reaction. Different amino acids exhibit varying degrees of reactivity towards reducing sugars, and the reaction’s outcome depends on the type and concentration of amino acids [4]. Lysine and arginine, which contain primary amino groups, are highly reactive towards reducing sugars and are frequently used in Maillard reaction studies to investigate reaction mechanisms and product formation [72]. In contrast, amino acids such as alanine, glycine, and proline are less reactive towards reducing sugars and contribute to the structural stability of proteins and are less likely to participate in the Maillard reaction [73]. The concentration of amino acids also affects the Maillard reaction’s rate and product formation. In general, a higher concentration of amino acids leads to faster reaction rates and higher product yields [74]. However, at high concentrations, amino acids can also compete with each other for reaction with reducing sugars, leading to the formation of unwanted byproducts [56].

The interactions between amino acids and reducing sugars can lead to the formation of different MRPs [4]. For example, the reaction between lysine and fructose can lead to the formation of a specific MRP: fructoselysine [75]. Another example is the reaction between glutamine and glucose, which can lead to the formation of an intermediate product: 5-hydroxymethylfurfural [76]. Additionally, the presence of other components such as lipids, minerals, and vitamins can also affect the rate and product formation of the Maillard reaction by catalyzing or inhibiting the reaction [77].

##### Lateral Secondary Amino Group of Proteins: Residues of Histidine

Histidinyl residues (His, H) contain a secondary amino group (R–NH–R′) that can participate in nucleophilic addition reactions which have been rarely described. One reason is the relatively low abundance of histidine residues in proteins, accounting for approximately 2.2% of amino acid residues [78]. While secondary amino groups are nucleophilic, the pKa of a typical secondary amino group is around 9. For histidinyl groups, however, the pKa is lower, approximately 6.8 [79], making them poorer nucleophiles. Consequently, the alkylation of histidine residues occurs less frequently than lysine residues, which have a higher pKa (~10) and are stronger nucleophiles.

**Figure 9:**
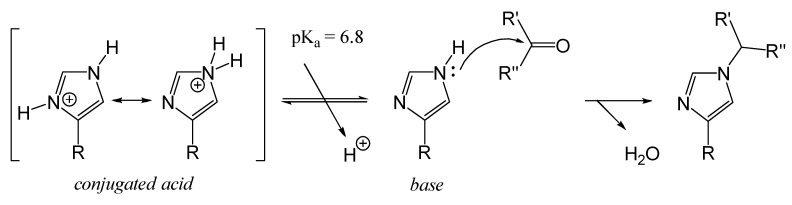
In the early stages of the Maillard reaction, the imidazole side chain of histidine can act as a nucleophile, attacking the electrophilic carbonyl carbon of reducing sugars. This nucleophilic addition leads to the formation of a Schiff base, which can undergo further rearrangements and reactions, contributing to the complex network of Maillard reaction products. Such interactions may result in the alkylation of the histidine residue, forming stable adducts that can influence the structure and function of proteins.

Histidinyl residues are utilized in bioconjugation strategies through alkylation reactions (Maillard reaction) to label proteins selectively, thereby avoiding the labeling of Cys, Lys, or Tyr residues [78]. Additionally, the reaction of histidine residues with secondary oxidation products, such as aldehydes like 4,5-epoxy-2-alkenals [25] or 4-hydroxynonenal [24], has been observed. This reaction is illustrated in Figure 9.

##### Lateral Guanidine Group of Proteins: Residues of Arginine

Arginyl residues (Arg, R) contain a guanidino group (H_2_N–C(=NH)–NH–R) and can participate in nucleophilic addition reactions which have been rarely described. This is mainly because the pKa value of the guanidinium group is very high, and the typical pH range of food systems (pH 5–7) is far from this value. The pKa of the guanidinium group in free arginine is approximately 13.8 [80]. In reality, the pKa of arginyl residues in proteins depends on the specific position of the residue within the protein structure and the local electronic microenvironment, with reported pKa values ranging from 11.5 to 15.0. A commonly accepted average pKa value is around 12 [81].

**Figure 10:**
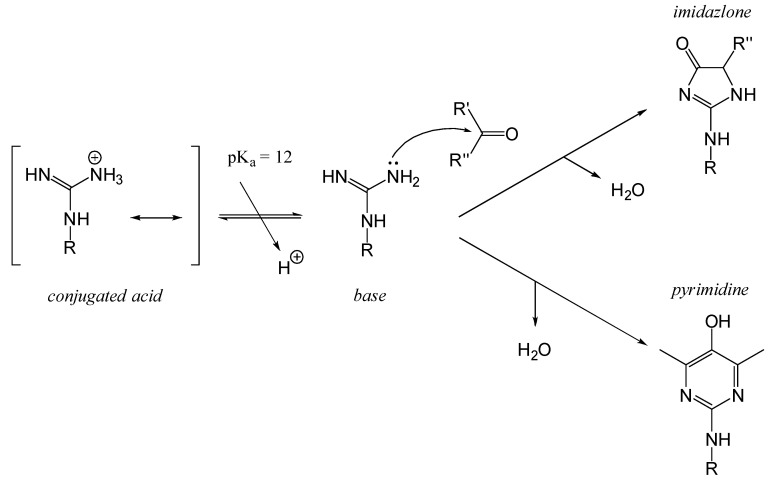
Nucleophilic addition of arginine residues to carbonyl compounds leading to the formation of imidazolone and pyrimidine adducts. The guanidino group of arginine reacts with reactive carbonyl species resulting in the formation of advanced glycation end-products (AGEs) like imidazolones and pyrimidines. These modifications can alter protein structure and function, contributing to changes in food quality and potential health implications. R′ = H or CH_2_OH.

**Figure 11:**
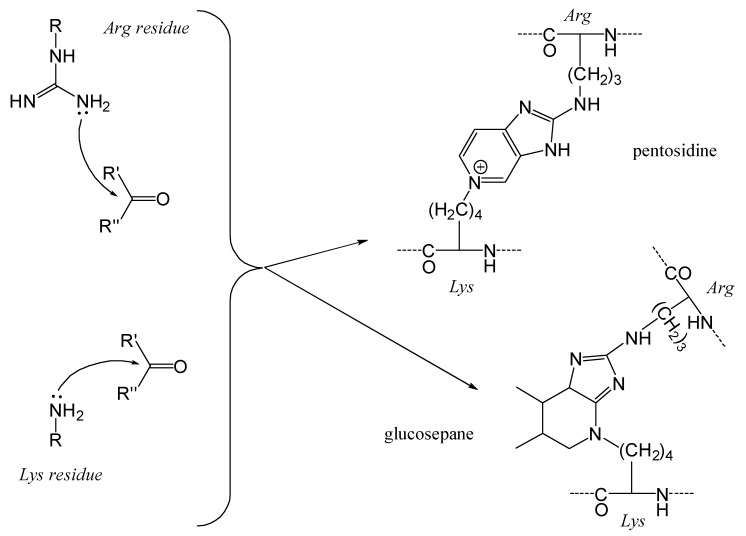
Nucleophilic addition of lysine and arginine residues to carbonyl compounds leading to protein cross-linking. The ε-amino group of lysine and the guanidino group of arginine react with reactive carbonyl species, resulting in the formation of advanced glycation end-products (AGEs) like pentosidine or glucosepane. These modifications can lead to protein cross-linking, altering protein structure and function, which may impact food quality and have potential health implications.

The MRPs involving arginyl residues include advanced glycation end-products (AGEs) and advanced lipoxidation end-products (ALEs), formed through reactions with carbonyl groups from lipid oxidation products. These include structures such as imidazolone and pyrimidine (Figure 10). Additionally, arginine and lysine residues can be cross-linked through the formation of compounds like pentosidine or glucosepane, leading to protein crosslinking, as illustrated in Figure 11 [82].

Maillard reactions are used to modify and functionalize proteins, such as pea proteins. The modification of arginine residues is considered a primary factor in altering the functional properties of proteins, comparable to the modification of lysine residues [83].

##### Lateral Primary Amino Group of Proteins; Lysine Residues

Lysyl residues (Lys, K) possess primary amino groups (R–NH_2_) and are extensively involved in nucleophilic addition reactions. An additional ε-amino group is also available, making lysine particularly reactive in Maillard-type reactions. In fact, reactions involving lysyl residues are the most widely, and almost exclusively, described in scientific literature. The pKa values of lysyl residues vary depending on their position within the three-dimensional structure of the protein. For example, in mammalian calmodulin, the pKa values of the seven lysyl residues range from 9.3 to 10.2 [84]. Lysyl residues located within the hydrophobic core of proteins can exhibit very low pKa values—as low as 5.3—due to the reduced polarity and polarizability of the environment compared to residues exposed to the aqueous phase. Thus, depending on the structural location, lysyl residues may have pKa values ranging from 5.3 to 9.3. In contrast, lysyl residues exposed to the aqueous phase generally exhibit consistent pKa values around 10.4 [85].

As expected, the reactive groups involved in Maillard reactions are typically located on the protein surface. Analysis of lysyl residue alkylation during Maillard reactions between lactoferrin or β-lactoglobulin and lactose shows preferential glycation of surface-exposed lysyl residues. In lactoferrin, Lys47 and Lys627 are particularly susceptible to glycation [86], while in α-lactalbumin and β-lactoglobulin, Lys5 (α-lactalbumin) and Lys47 (predominantly), as well as Lys138 and Lys141 (β-lactoglobulin), are preferential sites of glycation [87]. These observations likely correlate with the surface exposure of these residues. Therefore, in subsequent discussions, a pKa value of 10.4 will be considered for lysyl groups that are accessible to water and available for reaction with carbonyl compounds in the aqueous phase.

Peptides and proteins contribute through their terminal α-amino groups and predominantly through ε-amino groups from lysine residues. The amino group content, when proteins are the substrate, depends on the primary structure of the protein. For instance, αs_1_-casein (a milk protein) contains 14 lysine residues among 199 aminoacyl residues, and αs_2_-casein contains 24 lysine residues among 207 aminoacyl residues [88]. In contrast, gluten proteins, such as gliadins and glutenins, are very poor in lysine and are mainly composed of glutamine, proline, phenylalanine, tyrosine, and glycine residues [89], which have side chains lacking amino functions. In these proteins, lysine residues account for less than 1% of aminoacyl residues [90,91].

##### Effect of the Type and Concentration of Reducing Sugars on Maillard Reaction

The type and concentration of reducing sugars also significantly influence the reaction rate and product formation of the Maillard reaction [56]. Different reducing sugars exhibit varying degrees of reactivity towards amino acids, and the choice of the reducing sugar affects the reaction outcome [55,92]. For example, fructose is more reactive towards amino acids than glucose, leading to a higher rate of Maillard reaction and formation of specific MRPs. The concentration of reducing sugars also affects the reaction rate and product formation. A higher concentration of reducing sugars leads to a faster reaction rate and higher product yields. However, at high concentrations, reducing sugars can also lead to the formation of unwanted byproducts such as acrylamide [93].

Common examples of reducing sugars include ribose, glucose, fructose, lactose, and maltose. Notably, sucrose is not classified as a reducing sugar and therefore does not participate directly in the Maillard reaction under standard conditions. Monosaccharides are predominantly present in their cyclic forms, while the carbonyl group exists only in the open-chain structure. In aqueous solutions, the open-chain (anomeric) form is in equilibrium with the cyclic structure. For example, in glucose, the cyclic pyranose form represents 99.9974% of the total glucose, with only 0.0026% existing as the open-chain form [94]. In contrast, sucrose does not form an open-chain structure and therefore lacks a reactive carbonyl group. As a result, sucrose is not a direct substrate for the Maillard reaction, unless it undergoes hydrolysis to yield glucose and fructose, which are reactive substrates. The addition of sucrose to formulations, intended to provide sweetness, generally does not promote the Maillard reaction under mild heating conditions. Conversely, to enhance the Maillard reaction, the addition of glucose or fructose (for sweet products) or lactose (to limit sweetness) is a more effective formulation strategy.

#### 3.5.2. Effect of Amino Acid and Reducing Sugar Content on Product Safety and Quality

The amino acid and reducing sugar content can significantly affect the safety and quality of MRPs. The Maillard reaction can lead to the formation of harmful compounds such as acrylamide, which is a carcinogen. Therefore, controlling the amino acid and reducing sugar content is crucial in regulating acrylamide formation while maintaining desirable product properties [4,95]. The amino acid and reducing sugar content can also affect product quality, with higher concentrations leading to a faster reaction rate and the formation of undesirable products, such as dark colors and off-flavors [56]. Lower concentrations, on the other hand, can lead to a slower reaction rate, leading to reduced color and aroma. The formation of polymers between amino acids and reducing sugars during the Maillard reaction can cause amino acid decomposition and ultimately generate in-situ ammonia [96]. The formation of mutagenic compounds is a major concern when it comes to the pyrolysis of Maillard polymers. Therefore, controlling amino acid and reducing sugar content is critical in maintaining product safety and quality while achieving the desired flavor and color development [2,97].

### 3.6. Vitamin C Degradation

**Figure 12:**
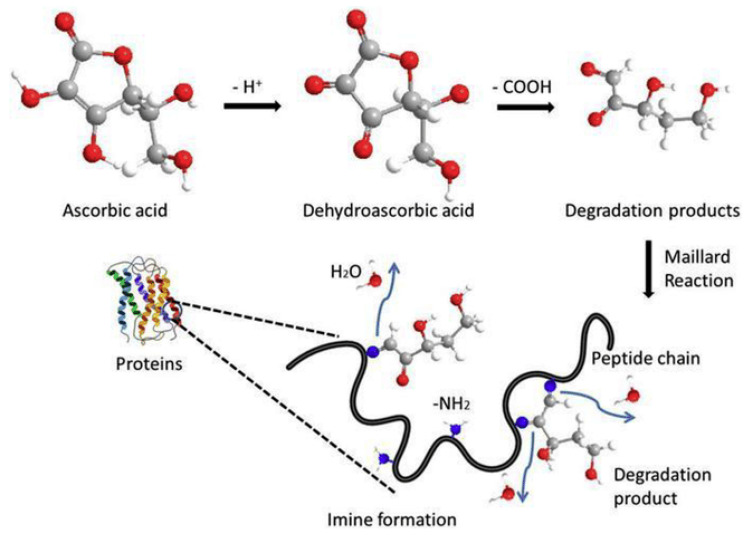
Diagram illustrating the proposed mechanism for the Maillard reaction between ascorbic acid (vitamin C) and whey protein isolate (WPI) [99].

From a structural perspective, ascorbic acid (vitamin C) is a reducing carbohydrate, making it actively involved in the Maillard reaction along with amino acids, peptides, and proteins. Vitamin C degradation occurs through Maillard reaction, where vitamin C reacts with amino acids, peptides, and proteins, leading to the formation of various end-products like carbonyl compounds, carboxylic acids, and amides (Figure 12). Maillard-induced degradation of vitamin C can result in browning of cut fruits, changes in food flavor, and potentially negative effects on health such as clouding of the lenses in the eyes and age-related loss of skin elasticity [98].

#### 3.6.1. Effect of the MRPs on Vitamin C Degradation

The Maillard reaction has a significant impact on the degradation of vitamin C, an essential nutrient in foods that is prone to deterioration under various circumstances like heat, pH fluctuations, and chemical processes [100]. This reaction occurs at high temperatures and in acidic environments, contributing to the breakdown of vitamin C. During the Maillard reaction, reactive compounds are produced, which can interact with vitamin C, leading to its degradation. The degree of vitamin C degradation is influenced by factors such as the duration, the intensity of the Maillard reaction, and the specific food type. Therefore, it is crucial to regulate the Maillard reaction to minimize vitamin C degradation while preserving the desired quality of the product [14].

The Maillard reaction not only impacts vitamin C degradation but can also give rise to various compounds that influence this process. Depending on the reaction conditions, products like melanoidins, furans, and pyrazines are formed. These compounds can interact with vitamin C, contributing to its degradation [56]. Specific MRPs, such as acrylamide, have been identified as factors that accelerate vitamin C degradation. Conversely, certain products from the Maillard reaction can scavenge free radicals, potentially mitigating their detrimental effects on vitamin C stability. This intricate interplay between MRPs and vitamin C degradation underscores the importance of meticulous consideration when developing food formulations [101].

#### 3.6.2. Effect of Vitamin C Degradation on Product Quality and Safety

As a vital antioxidant, vitamin C plays a crucial role in preserving the stability and nutritional integrity of food items. Its degradation can result in diminished antioxidant properties and shortened product shelf-life [14]. Moreover, the breakdown of vitamin C can give rise to the formation of detrimental compounds like diketopiperazines, posing potential risks to product safety. Hence, effective management of vitamin C degradation is paramount for preserving the quality and safety standards of food products [11]. Given the impact of the Maillard reaction on vitamin C degradation, meticulous control over the reaction’s processes and conditions is essential for safeguarding the nutritional value and safety of food products. On the other hand, and to minimize the formation of harmful compounds, additives like ascorbic acid and cysteine are used which were shown to inhibit the formation of acrylamide during the Maillard reaction [29].

## 4. S-Maillard Reaction

The Maillard reaction, first described by Louis-Camille Maillard in 1912, is a complex series of non-enzymatic browning reactions that occur during thermal food processing. Particularly important are the sulfur-containing compounds, such as cysteine, which drastically influence flavor, aroma, and color formation. Recent advances have shown that the presence of thiol groups can significantly alter the pathways and outcomes of the Maillard reaction.

Cysteinyl residues (Cys, C) contain thiolate groups (R–S^−^) that can participate in nucleophilic addition reactions which have not been frequently reported in the literature. The pKa value of the sulfhydryl/thiolate acid–base couple can vary widely [102], ranging, for example, from 6.7 to 9 for two cysteinyl residues within the same protein [103]. The position of the Cys residue within the protein structure and the electronic environment surrounding the sulfhydryl/thiolate group have a major influence on its pKa value. Factors such as temperature and the experimental methods used can also affect the pKa measurements. For instance, the pKa value of a cysteine (thiol) residue in a peptide, without the influence of a folded protein structure, has been estimated between 7.57 and 7.68 [104]. In contrast, the pKa value of a thiol group in β-lactoglobulin has been reported to be 9.35 [105].

**Figure 13:**
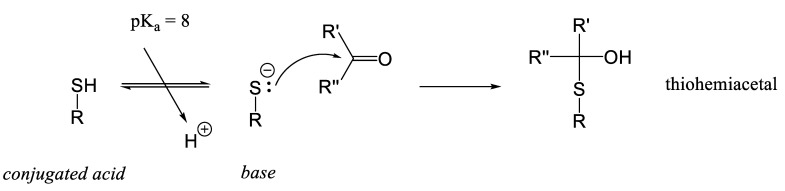
Nucleophilic addition of cysteine residues to carbonyl compounds during the early stages of the Maillard reaction. The thiol group of cysteine reacts with reactive carbonyl species, such as reducing sugars, leading to the formation of thiohemiacetal intermediates. These initial adducts can undergo further transformations, contributing to the development of flavor and color in food products.

Cysteine can react with carbonyl compounds in a Maillard-type reaction. Specifically, cysteine reacts with glyoxal (a dialdehyde) to form a thiohemiacetal, which can subsequently undergo a Cannizzaro rearrangement to yield S-carboxymethylcysteine [82]. The reaction between thiol groups and carbonyls, leading to the formation of thiohemiacetals as the initial product, was first described earlier (Figure 13) [106].

The addition of thiol compounds, such as garlic-derived thiols or cysteine, is used to limit the consequences of the Maillard reaction by ‘trapping’ hydroxymethylfurfural, a key intermediate produced during the reaction. The competitive reaction between cysteine and hydroxymethylfurfural (a carbonyl compound) reduces the interaction between lysyl residues and hydroxymethylfurfural, thereby preventing the formation of melanoidins and other advanced glycation end-products [107]. The nucleophilic addition of thiol groups to the aldehyde involves one mole of hydroxymethylfurfural and two moles of cysteine, resulting in the formation of 1-dicysteinethioacetal-5-hydroxymethylfurfural.

Cysteinyl residues are also involved in sugar-independent pathways, reacting with lysyl or other cysteinyl residues which contributes to protein crosslinking. Cysteine residues (as well as serine residues) can undergo β-elimination, resulting in the formation of dehydroalanine residues. The latter can then react with lysine or cysteine residues, in competition with the Maillard reaction, particularly during the heating of milk proteins (caseins and whey proteins) in the presence of lactose. These reactions lead to the formation of lanthionine (Ala–Cys ε-bond) and lysinoalanine (Ala–Lys ε-bond) residues, both of which promote protein crosslinking [108].

When the nucleophile is a thiolate group rather than an amino group, its content in proteins is very low. Most cysteine residues in globular proteins form disulfide bonds (R–S–S–R), and free cysteinyl residues with thiolate functions are rare. For example, β-lactoglobulin has only one free thiolate group among 162 aminoacyl residues [88], while ovalbumin from egg white contains four free thiol groups among 385 aminoacyl residues [109,110].

A significant discovery by Noda et al. [111] was the identification of a new Maillard pigment, pyrrolothiazolate, formed from L-cysteine and D-glucose. This compound displayed strong antioxidant properties, indicating that not all Maillard pigments are merely by-products but can also contribute functional benefits to foods.

While the Maillard reaction enhances flavor, it also produces harmful compounds like acrylamide. Augustine and Bent (2022) [101] emphasized that sulfur-containing compounds, particularly thiols, are efficient inhibitors of acrylamide formation. However, their effectiveness may come at the cost of altering the sensory properties of the final product.

The dual role of cysteine in the Maillard reaction was highlighted by Sun et al. [112]. Their research revealed that the thiol group in cysteine inhibits color formation with dihydroxyacetone, whereas the amino group enhances browning. This discovery is particularly useful in applications like sunless tanning, where controlled color development is desired.

Sulfur compounds play a major role in flavor development. Research by Zhang et al. [113] explored how the combination of vegetable oils and a D-xylose/L-cysteine system could produce rich meaty aromas without the use of animal-derived ingredients. Similarly, Yu et al. [114] showed that sulfur-containing volatile compounds, such as thiophenes and thiazoles, generated from reactions between L-ascorbic acid and cysteine, are crucial for delivering roasted and meaty flavors.

Billaud et al. [115] reported that certain MRPs derived from thiol compounds effectively inhibit enzymatic browning in fruits and vegetables. The most potent inhibitory products were obtained when combining glucose with cysteine or glutathione, offering an appealing natural alternative to sulfites.

In a seminal study, Hofmann and Schieberle (2002) [116] investigated the chemical mechanisms underlying aroma staling in coffee beverages. They demonstrated that odor-active thiols such as 2-furfurylthiol and 2-methyl-3-furanthiol, essential for the characteristic sulfury–roasty aroma, are rapidly lost when coffee is kept warm. Their experiments revealed that thiols are not simply oxidized but become covalently bound to coffee melanoidins via Maillard-derived pyrazinium intermediates (specifically, CROSSPY-type radicals). For instance, the concentration of 2-furfurylthiol decreased by a factor of 16 upon interaction with melanoidins. This binding significantly diminishes the overall sulfury perception in coffee and is considered a major cause of flavor deterioration during storage.

Recent studies shed new light on the multifaceted role of sulfur-containing compounds in the Maillard reaction. From promoting antioxidant pigment formation to enhancing flavor or even controlling toxic by-products, thiols and related sulfur molecules are invaluable tools in food chemistry. Furthermore, Hofmann’s findings on the binding of thiols to melanoidins offer critical insight into aroma stability, an aspect crucial not only for coffee but also for a broader range of thermally processed foods. Future work will likely focus on controlling these reactions to optimize food quality and shelf life.

## 5. Advantages and Beneficial Nutritional Effects of Maillard Reaction

The Maillard reaction is a highly important process in the food industry that produces many of the pleasant attributes we associate with various foods. Some of its benefits include:

### 5.1. Improvement in Color, Odor, and Flavor

Because of the physical and chemical characteristics of lactose, it is frequently used in the food sector. When lactose reacts with protein and amino acids in food, it can provide a variety of foods, including dairy products, candies, baked goods, sauces, quick drinks, beer, etc., an appealing look and flavor. The interaction of lactose with milk protein produces most of the aromas in some heat-treated dairy products, such as ultra-heat treated (UTH) milk, milk powders, cheese, etc. According to research done by Xiang et al. [5], MRPs like 2-acetylfuran, furfuran, hydroxymethylfurfural, furtol, and furfuryl alcohol are the primary source of these scents. The aroma in milk powder is linked to MRPs, including aldehydes, furaneol, and maltol. Additionally, food flavor could be enhanced by the Maillard reaction. Cantonese sausages’ stickiness might be decreased and their chewiness could be increased by the MRPs created by the combination of sugars and amino acids, which would enhance the sausages’ flavor.

The Maillard reaction produces a variety of chemicals, including pyrazines, furans, and aldehydes, which contribute to the pleasant flavor of the food [13]. Furthermore, improved flavor can have a beneficial impact on nutrition. Foods that are more appealing and flavorful are more likely to be consumed in larger quantities, ensuring that people get enough nutrients. This is particularly beneficial for people with dietary limitations or requirements, as it may encourage them to add nutrients to their diet [4].

### 5.2. Shelf-Life Extension

For several food products, the Maillard reaction is essential to extend their shelf-life. Although improving flavors, smells, and appearance is the main goal of the Maillard reaction, it also produces chemicals with antibacterial qualities. These antimicrobial substances, like furans, make the environment unfavorable for bacteria that cause food spoiling and prevent their proliferation, therefore, slow down the spoiling process and extend the freshness and shelf-life of food products [2,4,117]. It is well recognized that microorganisms, such as molds, yeasts, and bacteria, are mostly responsible for food spoilage. The growth and multiplication of these organisms can result in unfavorable alterations to the texture, flavor, and appearance of food, making it unfit for human ingestion [118].

Research is still ongoing to determine the methods by which the chemicals involved in the Maillard reaction exert their antimicrobial properties. These substances could damage cell membranes, block enzymes required for microbial growth, and interfere with metabolic processes in microorganisms. Moreover, they might have antioxidant properties which prevent oxidative damage and the development of spoilage microorganisms [119].

The food product and its composition have a significant impact on how well the Maillard reaction, and its resulting antimicrobial chemicals, enhance shelf-life. Also, shelf-life can be affected by several variables, including pH, water activity, and the presence of other reactants. For instance, since acidic environments are less favorable to microbial growth, lower pH values might increase the antimicrobial effect of the Maillard reaction [120]. On the other hand, food producers frequently use the antibacterial characteristics of Maillard reaction chemicals in various processed and packaged food products. These molecules have the potential to operate as natural preservatives, allowing for a longer shelf life without the use of synthetic additives. This not only satisfies customer demand for clean label products but also adds another layer of defense against microbial deterioration [119]. In contrast, while MRPs may suppress spoiling microorganisms, they may not be effective against pathogenic germs that can cause foodborne diseases. As a result, adequate food safety standards, such as optimal temperature storage and adherence to hygiene practices during food preparation, remain critical in maintaining the safety of food products. Also, MRPs can help inhibit oxidative deterioration, extend shelf life, and improve overall food quality [121].

### 5.3. Increased Nutrient Density and Bioavailability

Aside from its role in flavor and aroma enhancement, the Maillard reaction can contribute to the densification and enhanced bioavailability of particular nutrients in food [2,4]. For example, amino acids and reducing sugars can create complexes that enhance mineral solubility and absorption, such as iron and zinc. These complexes are more readily absorbed by the body, boosting nutritional bioavailability [4]. Furthermore, the Maillard reaction can produce compounds with health benefits, such as antioxidants like melanoidins and some phenolic compounds [122]. The melanoidins are brown-colored polymers generated during the Maillard reaction; they were found to be potent antioxidants, and can scavenge free radicals, prevent lipid peroxidation, and protect the cells from damage caused by oxidation. These antioxidant properties may aid in the prevention of chronic diseases such as cardiovascular disease, cancer, and neurological disorders [123]. In addition, depending on the amino acids and reducing sugars involved, the Maillard reaction can result in the synthesis of a variety of phenolic compounds who also possess antioxidant capacity; they are known to scavenge free radicals, chelate metal ions, and inhibit certain oxidizing enzymes. These phenolic substances may exhibit more potent antioxidant activity than their precursors [124]. It is important to note that the Maillard reaction’s effect on nutrient density and bioavailability varies based on parameters such as cooking temperature, time, and ingredients. Overcooking or high heat can cause nutrients’ breakdown or the development of potentially hazardous chemicals. Therefore, to preserve maximal nutritional density and bioavailability, it is critical to use suitable cooking procedures [12].

An example of increased bioavailability is represented by the availability increase of antioxidants, such as phenolic compounds and flavonoids, which add to the body’s ability to neutralize damaging free radicals. The Maillard reaction can result in the production of antioxidant compounds as well as the degradation of bigger antioxidants into smaller, more absorbable forms [125].

### 5.4. Improved Protein Digestibility

Proteins are essential macronutrients involved in tissue development and repair, enzymatic synthesizing, and the manufacturing of hormones. Certain proteins, on the other hand, may be difficult for the body to properly break down and absorb effectively. Protein digestibility improves when it undergoes the Maillard process [68]. Protein structure can be altered by the chemical changes that occur during the Maillard reaction, making them more disposed to enzymatic degradation by digestive enzymes. This improves the body’s digestion and absorption of amino acids. This is particularly beneficial for people with poor digestion, such as the elderly or those suffering from gastrointestinal diseases [34].

## 6. Disadvantages of Maillard Reaction

The products of the Maillard reaction can be beneficial to human health, but they can also be harmful. The principal negative effects of the Maillard reaction are as follows:

### 6.1. Elimination of Important Amino Acids and Limiting Their Bioavailability

The loss of vital amino acids during food preparation and cooking is one of the potential downsides of the Maillard reaction. Amino acids are the building blocks of proteins, and they are required for many biological activities in the body, such as tissue repair, enzyme synthesis, and hormone manufacturing. When amino acids interact with reducing sugars throughout the Maillard reaction, they may undergo chemical changes, making them less available for protein synthesis or other critical functions [4]. As previously shown, amino acids undergo glycation via the Maillard reaction, where they react with reducing sugars to create advanced glycation end-products. While advanced glycation end-products contribute to the pleasant colors and flavors of cooked foods, they can also affect amino acid absorption and functionality [6]. The production of advanced glycation end-products might lead to a decrease in protein digestibility and efficiency. In fact, advanced glycation end-products can reduce the availability of proteins for absorption in the gastrointestinal system by inhibiting the activity of digestive enzymes that break them down. As a result, the capacity to obtain and utilize the amino acids required for optimum biological activities may be compromised [6]. The DIAAS (digestible indispensable amino acid score), the only nutritional quality parameter for protein recognized by the FAO, is decreased by Maillard reaction due to loss of lysine by Maillard reaction (irreversible alkylation of lysine) [126,127,128].

Furthermore, AGE accumulation in the body has been related to a variety of health problems, including diabetes, chronic renal disease, and coronary artery disease. Advanced glycation end-products lead to inflammation and oxidative stress, both of which are linked to the advancement of various diseases. The Maillard reaction may indirectly lead to an increased risk of developing certain health problems by restricting the availability and functionality of essential amino acids.

### 6.2. Formation of Potentially Harmful Substances

Although known for its capacity to enhance flavors and fragrances, the Maillard reaction can also result in the creation of potentially hazardous chemicals during food processing and cooking [4]. Acrylamide, a chemical molecule recognized as a possible human carcinogen by the International Agency for Research on Cancer (IARC), is formed during high heat cooking such as frying, baking, or roasting by a reaction between the amino acid asparagine and reducing sugars such as glucose and fructose (Figure 5). Carbohydrate-rich foods with low moisture content, such as potatoes, coffee, and crispy snacks, are especially prone to acrylamide formation by the Maillard reaction [7]. In addition, as previously shown in Figure 5, HMF is formed as a product of the Maillard reaction. While HMF is believed to possess certain beneficial biological properties, such as antioxidant activity and the ability to inhibit red blood cell sickling, it is metabolized in humans to 5-sulfoxymethylfurfural (SMF) and 5-hydroxymethyl-2-furoic acid (HMFA). SMF can form adducts with DNA or proteins, and toxicology studies in rodents have showed potential genotoxic and carcinogenic effects [30]. Moreover, Furan is detected in canned and jarred foods and baby food. It is categorized as possibly carcinogenic to humans by the IARC. One of the pathways of its formation is the Maillard reaction [129]. Other potentially dangerous compounds produced by the Maillard reaction include heterocyclic amines (HCAs) (Table 1). HCAs are generated when amino acids, creatinine, and sugars react at high temperatures, generally when meat, poultry, or fish are cooked [130]. These substances have been linked to an increased risk of cancer, including colorectal, stomach, and pancreatic cancer. In addition, N-Carboxymethyllysine (CML), another MRP, is also associated with multiple pathological conditions. Several mechanisms have been described by which CML can cause tissue damage. It was found to trigger inflammatory reactions, hyperglycemia, hyperlipidemia and enhanced oxidative stress, a typical metabolic profile in obesity and obesity-related complications [131].

Acrylamide and furans are heat-induced toxicants that pose significant toxicological concerns due to their genotoxic and carcinogenic potential. Acrylamide, formed during high-temperature processing via the Maillard reaction, is metabolized to glycidamide, a reactive epoxide capable of forming DNA adducts [132,133]. Glycidamide has been linked to neurotoxicity and carcinogenesis in rodent models, with estimated dietary exposures ranging from 0.3 to 1.0 µg/kg bw/day in humans [134]. In contrast, furan is primarily hepatotoxic, metabolized by cytochrome P450 enzymes to the reactive intermediate cis-2-butene-1,4-dial, which can bind to nucleophilic sites in proteins and DNA [135]. Chronic exposure to furan has been associated with hepatocellular adenomas and carcinomas in rats, with a BMDL10 (benchmark dose lower confidence limit) for cancer of approximately 0.064 mg/kg bw/day [136]. Both compounds exhibit dose-dependent toxicity, and quantitative risk assessments underscore the need for mitigation in thermally processed foods [137,138].

### 6.3. Nutrient Loss

The Maillard reaction can cause nutrient loss, especially in heat-sensitive vitamins such as vitamin C and thiamin [4]. Vitamin C, commonly known as ascorbic acid, is a necessary nutrient that serves as an antioxidant and aids in collagen formation, iron absorption, and immunological functions. Vitamin C is highly heat sensitive and easily destroyed during cooking, and this could be caused by the Maillard reaction since high temperatures are required for the mechanism to occur. High temperatures and extensive cooking times associated with this reaction might cause vitamin C breakage and loss [8].

Thiamin, often known as vitamin B1, is another heat-sensitive vitamin that can be affected by heating and cooking techniques that cause the Maillard reaction. Thiamin is essential for energy metabolism and nervous system functions [139]. Furthermore, the Maillard process can produce complex molecules that trap lysine, an essential amino acid, thus reducing the overall nutritional value of food. Lysine is required for protein synthesis and is a component of many compounds in the body [34].

### 6.4. Browning Restrictions

Excessive browning can produce harsh or scorched flavors, making the meal unpleasant or even inedible to consumers. Brown pigment, like melanoidins, that are responsible for the pleasant scent and flavor associated with well-cooked foods, are among the chemicals produced by the Maillard reaction [140]. While browning is usually desired, it can occur when foods is cooked at extremely high temperatures or for a prolonged period. This might result in the formation of bitter and unpleasant flavors, which can overshadow the dish’s intended flavor character. The level of acrylamide, the previously stated potentially dangerous chemical, can also increase in severely browned and overdone foods [141].

### 6.5. Difficulty to Regulate

The Maillard reaction is a complex chemical process that can be difficult to control due to a variety of reasons, for instance, the pH of the food or cooking environment, which is an important factor that controls the Maillard reaction [2]. On the other hand, higher temperatures can speed up the reaction, causing it to brown and produce flavor faster. However, precisely regulating the temperature becomes difficult, especially during cooking procedures where heat distribution may not be consistent. Temperature variations across the cooking surface can cause uneven browning and degrade the overall quality of the food [22]. In addition, the presence of water can speed up the reaction, and the amount of moisture in the meal can influence the rate of browning. Controlling the water content of foods while cooking can be tricky since different components have different moisture levels and heating behavior [22]. Furthermore, the duration of the Maillard reaction is an important consideration. Depending on the cooking time, the reaction may progress differently, affecting the extent of browning and taste development. Achieving the proper level of browning without going too far becomes difficult, since prolonged heat exposure can result in excessive browning and unwanted flavors [22].

## 7. Mitigation of Maillard Reaction Products

The MR and its products were found to possess many biological activities like antioxidant [142,143,144], antimicrobial [117,142,143], antihypertensive, α-glucosidase inhibitory, antihypertensive, antiproliferative [144,145], and anti-inflammatory [146]. Also, MRPs were found to influence the composition of the intestinal microbiota in both humans and rats; they might be beneficial for the maintenance of intestinal health [147,148]. In addition, Maillard reactions can sometimes have beneficial effects by altering allergen epitopes and lowering their allergenic potential. However, they may also worsen allergic reactions by modifying epitope motifs or creating new allergenic structures (neoallergens) [149].

In contrast, a higher dietary intake of these compounds has been associated with an increased risk of pathological conditions such as diabetes, cancer, chronic heart failure, Parkinson’s disease, and Alzheimer’s disease [56]. Therefore, implementing strategies to reduce or prevent their formation in heat-processed foods is crucial [150,151,152]. Consequently, various mitigation techniques have been studied to limit their occurrence, like phenolic compounds, food additives, thermal and non-thermal techniques, enzymes and encapsulation of metal ions.

### 7.1. Use of Additives

A study by Zhang et al. [153] reported that flavonoids are effective in reducing acrylamide formation. Constantinou and Koutsidis [154] showed that epicatechin reduced by 25–75% acrylamide content. Bhuiyan et al. [155] showed that quercetin acted as an effective inhibitor of advanced glycation end-products (AGEs) formation. Similarly, Culetu et al. [156] used theanine and polyphenol-enriched fractions derived from tea dust to inhibit fluorescent AGE formation in a bread model system. Moreover, Shen et al. [157] reported that resveratrol exhibited a strong inhibitory effect on AGE formation. Several bioactive compounds have been investigated for their potential to inhibit the formation of advanced glycation end-products (AGEs) like chlorogenic acid, and epigallocatechin gallate, extracted from black bitter plum [158], flavonoids [159], alkaloids [160], polysaccharides [161], hawthorn-derived polysaccharides [162], and terpenoids [163]. Studies have explored the use of natural product extracts and spices with antioxidant properties to reduce the formation of heterocyclic amines (HAAs). Plant-based extracts have demonstrated inhibitory effects on HAA formation, including ethanol-extracted nutmeg [164], blueberry, and propolis extracts [165], *Portulaca oleracea* extract [166], *Sonchus oleraceus* extract [167], olive leaf extract [168], *Adinandra nitida* leaf extract [158], avocado peel extract [169], hawthorn extract [170], chrysanthemum extract [171], raspberry, blueberry, and strawberry extracts [172], and blueberries, cherries, and grapes [173]. These plant sources are rich in natural antioxidants that can inhibit HAA formation [174]. Moreover, several spices have been studied for their HAA mitigation, including turmeric, cloves, cinnamon, rosemary, black pepper, garlic, black cumin, and chili pepper. The authors of [175] reported that garlic, black pepper, and chili pepper significantly inhibited HAA formation during sausage processing. To mitigate acrylamide (AA) formation, various inhibitors such as vitamins, natural extracts, amino acids, and other compounds have been studied. Arámbula Villa et al. [176] showed that soaking potato slices in magnesium chloride and calcium chloride solutions reduced AA levels by 74% and 67%, respectively. Additionally, glutathione was found to reduce AA formation [177]. Several natural extracts have also been used to mitigate AA formation, including procyanidins [178,179], tomato, and pomegranate extracts [180], bamboo leaf, and buckwheat extracts [166], as well as 1% ginger powder [181]. The formation of 5-hydroxymethylfurfural (HMF) during food processing can be effectively reduced through the incorporation of additives. Zhu et al. [182] reported that the addition of 2% histidine to cookie formulations inhibited HMF formation by up to 90%. Phenolic compounds have also shown significant potential in reducing HMF levels. Pedreschi et al. [183] demonstrated that polyphenolic extracts from *Caesalpinia spinosa* (tara pods) effectively decreased HMF content in bread. Similarly, Zhang and An [184] found that quercetin, when added to potato chips and bread, reduced HMF formation by 50% to 86%. In another study, Abrantes et al. [185] reported that gallic acid also contributed to a reduction in 5-HMF formation.

### 7.2. Thermal and Non-Thermal Techniques

Recent advancements in food processing have led to the development of both thermal and non-thermal technologies that aim to improve product quality and to mitigate the formation of MRPs. Variables such as processing time, pH, temperature, concentration and availability of reactants, water activity, as well as the presence of metal ions, can be investigated to mitigate MRPs [43]. Innovative thermal techniques, such as vacuum frying, vacuum drying, and vacuum roasting, offer improved control over MRPs formation as compared to conventional heat treatments. In addition, several non-thermal methods like pulsed electric fields (PEF), high-pressure processing, ohmic heating, and infrared heating are investigated for the same reason. PEF technology has gained attention for its ability to process food with minimal thermal processing effect. The frequency, width, and polarity of the electric pulses significantly influence the extent of MRPs formation [2,62,186].

#### 7.2.1. Microwave Heating

Microwave heating is an energy-efficient and cost-effective technique that contributes to the reduction of acrylamide (AA) formation in food products. This method effectively lowers moisture content, reduces frying temperature, and shortens frying duration, factors that limit AA production [187]. Sansano et al. [188] showed that microwave-assisted frying can reduce acrylamide levels by 37–83% compared to traditional deep-frying methods. Furthermore, microwave processing has been shown to significantly reduce processing time and energy consumption while maintaining the nutritional quality of food. It also serves as a promising method to limit the formation of harmful compounds such as acrylamide during frying [189].

#### 7.2.2. High Pressure Processing

High pressure processing (HPP) is a non-thermal preservation method that effectively limits the formation of thermally induced compounds in food. Yi et al. [190] reported that HPP-treated apple juice contained fewer heat-induced compounds compared to traditionally pasteurized juice. One of the main advantages of HPP is its low processing temperature, which causes minimal chemical alterations in food matrices [62]. Interestingly, Avila Ruiz et al. [191] found that combining high pressure (700 MPa) with elevated temperatures (123 °C) effectively suppressed the Maillard reaction and preserved the color in whey protein–sugar systems.

#### 7.2.3. Ohmic Heating

Ohmic heating involves the direct conversion of electrical energy into thermal energy as current flows through food, which acts as an electrical resistor [192]. This technology enables rapid and uniform heating, leading to reduced formation of MRPs [2]. Several studies have demonstrated that ohmic heating results in lower MRP levels than conventional thermal methods [62,192,193]. Pires et al. [194] reported a significant reduction in the formation of HMF and related degradation products using this method.

#### 7.2.4. Air Frying and Vacuum Frying

Air frying operates at lower temperatures than conventional frying and has been shown to effectively reduce acrylamide formation while maintaining desirable sensory characteristics [195]. Verma et al. [196] observed that both air frying and vacuum frying significantly decreased acrylamide and HMF concentrations in French fries, with inhibition rates of 78.7% and 81.14%, respectively, in potato chips. Belkova et al. [197] reported a 98% reduction in acrylamide formation compared to traditional frying, along with decreased pyrazine levels. According to Devseren et al. [198], optimal vacuum frying conditions (124.39 °C for 8.36 min) produced chips with improved color, flavor, and minimal acrylamide content.

### 7.3. Enzymatic Treatment

The use of enzymes, particularly asparaginase, is among the most promising strategies for acrylamide reduction. Asparaginase catalyzes the hydrolysis of asparagine into aspartic acid, thus preventing acrylamide formation during high-temperature processing [141]. Luo et al. [199] used 1 U/mL glucose oxidase to treat melon juice at 35 °C for 180 min, observing reduced degradation of key aroma compounds associated with Maillard and oxidation reactions. The effectiveness of asparaginase in acrylamide mitigation has been validated across various food systems [200,201].

### 7.4. Fermentation

Fermentation also offers potential for mitigating harmful Maillard reaction products. Lemos et al. [202] reported that fermentation can convert 5-hydroxymethylfurfural (HMF) into its less harmful alcohol derivative, hydroxymethylfurfuryl alcohol (HMF alcohol), thereby reducing HMF levels in food matrices.

## 8. Food Industrial Applications of the Maillard Reaction

The Maillard reaction is extensively utilized in the agri-food industry for various food processing applications to enhance flavors and colors desired by consumers. This reaction is crucial in food productions like coffee and bakery products with appealing color and flavor profiles [56].

**Figure 14:**
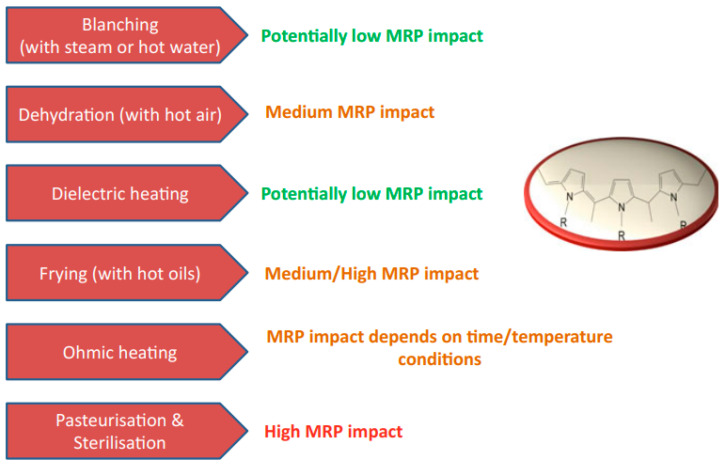
The influence of heat treatments in food processing on MRP formation [204].

The Maillard reaction occurs in food processing at high temperatures such as baking, roasting, frying or others as well as extrusion or long storage [203]. It is of interest in the manufacturing of various food items such as bread, cakes, meat, fish, potato-based products, cocoa, coffee, dairy desserts, etc. to enhance their color, texture, and flavor, making them more appealing to consumers. However, the Maillard reaction can have undesirable outcomes in certain products such as in milk and fruit juices, due to the development of an unappealing brown color [56]. The influence of different heat treatments techniques in food processing on the MRPs formation is of high importance in the food industry. The effect of different techniques is shown in Figure 14. Blanching, a low-thermal treatment, is primarily used to stop enzymatic activity and inhibit microbial proliferation in freshly harvested foods. This process often yields low MRPs levels, notably in the case of acrylamide formation in French fries. On the other hand, dehydration removes water using heat. As a result, MRPs are frequently found in dehydrated products like orange juice, raisins, onions, garlic, etc. On the other side, frying techniques seem to be closely linked to the production of MRPs, particularly acrylamide. Boiling processes are found to favor the Maillard reaction, particularly in products such as beers, milk, and rice wines. The effect of ohmic heating on the production of MRPs in foods is primarily determined by the time/temperature combinations used. Pasteurization and sterilization are known to influence MRP output in different foods [204].

### 8.1. Soybean Processing

Soybeans are used in many applications such as in cooking oil, granules, grits productions, and others. They contain omega-3 fatty acids, known to have beneficial effects on the cardiovascular system and to possess anticancer activity [205,206]. Soybeans can be heated using three different techniques: microwave heating, extrusion heating and infrared heating.

There are two methods for applying microwave heating treatment. The first, known as short-time heating, would acquire 1–2 min, while the second would acquire 3–5 min and is known as long-time heating [205,207]. When soybeans are heated in the microwave in the short-time method, acrylamide is abundantly formed. On the other hand, if it is heated for a long time, the formation of acrylamide decreases [187,205]. In addition, the formation of acrylamide under extrusion and infrared heating varies greatly depending on the temperature and the time: Increasing the temperature and prolonging the application time results in higher formation of acrylamide. In addition, HMF production is time and temperature dependent [208]. When the temperature and time are raised, HMF production increases. Also, microwave heating treatment conditions were found to improve the antioxidant capacity of soybeans [205,208].

### 8.2. Milk Processing

Milk, consumed across the world, is a complex mixture containing fats, sugars, and proteins. In milk processing, there are primarily two types of heat treatment methods: ultra-high temperature (UHT) treatment and conventional sterilization [205,209]. As a result of heat treatment, MRPs can be formed. A reaction may occur between milk lactose and the amino group of lysine, which causes its loss in milk. This affects the nutritional value of milk. The degree of lysine loss is proportional to the heating temperature used [5,205]. In addition, the MRPs may have ion-chelating capacity, which decreases mineral bioavailability. Solubility loss in milk protein concentrate powder is a relatively new phrase. This solubility loss is said to be caused by the Maillard reaction [5,205].

Milk contains, approximately, 80% casein and 20% whey protein, along with high levels of lactose; therefore, MRPs are produced when processed into products such as UHT milk and milk powder, especially during storage [210]. Li et al. [211] investigated the impact of different heat treatments on MRP formation in whole milk powder (WMP) during storage. Their findings showed that low-heat WMP had higher furosine levels, whereas high-heat WMP exhibited elevated amounts of HMF, Nε-(carboxyethyl)lysine (CEL), and CML. Similarly, Nielsen et al. [108] observed an increase in MRPs like CEL and furosine with heat treatment. They also noted that dehydroalanine derivatives, including lanthionine and lysinoalanine, increased with heat in both casein and whey protein isolates, regardless of the presence of lactose.

### 8.3. Meat Processing

Maillard reaction is used in meat processing to change the color and flavor of the meat. In meat preparation, elevated temperatures may increase the amount of potentially toxic heterocyclic amine (HCA) [4,205]. Food frying and boiling increase HCAs, and these HCAs are responsible for various flavors and tastes. In fact, it has been discovered that several heterocyclic compounds such as pyrazine, oxazole, and thiazoles are responsible for the roasted flavor. Pyrazine content increases during high heat treatment like grilling [4,205].

Li et al. [211] reported that during the production of dry fermented meat products, amino acids play a key role in generating volatile compounds through Maillard reaction, a process further enhanced by microbial activity. Amino acids, along with reducing sugars, fats, and thiamine, serve as essential flavor precursors in meat, contributing to its distinctive aroma when subjected to heat treatment. Dong et al. [212] found that ultrasonic-assisted heat treatment promotes amino acid–sugar cross-linking while reducing the bitterness of MRPs, suggesting its potential for enhancing Maillard-derived flavors. This technique also holds promise for the utilization of mussel meat in developing flavor enhancers and functional foods. Tavares et al. [213] observed a lower formation of MRPs during the early and advanced stages of boiling hairtail fillets, compared to baking and frying. Specifically, CML and furosine levels in these fillets ranged from non-detectable to 4.24 mg/100 g protein and 4.25 to 20.95 mg/100 g protein, respectively. Furthermore, Tamanna and Mahmood [4] highlighted that pan-frying leads to higher MRP formation compared to other cooking methods such as charcoal grilling, deep frying, roasting, microwaving, or boiling.

### 8.4. Brewing Industry

Flavor is a fundamental factor in determining beer quality and a critical component of consumer acceptance. Many variables, such as yeast strain, malt type, and fermentation parameters, can influence the distinctive flavor profile of beer, whereas the perceived flavor of beer is attributed to the specific ratios of a variety of compounds, such as higher alcohols and esters, rather than the impact of a single compound. Volatile (aldehydes, ketones) and non-volatile compounds (organic salts, sugars, amino acids, and organic/inorganic acids) both contribute to the organoleptic profile of beer, but higher alcohols and esters are regarded as the most important flavor active compounds [214]. Malting barley kernels produce enzymes that break down high molecular weight nutrients, such as starch granules and insoluble proteins, into lower molecular weight components that are substrates for fermentation [215]. Malt kilning, which is the drying and heating of the germinated malt, creates the right conditions for the Maillard reaction. Due to the low moisture level at the end, kilning is controlled by maltsters to produce the numerous color and flavor combinations that brewers use to create distinct beer styles. Depending on the temperature, they may obtain green malt, caramelized malt, chocolate malt and black malts. These are significantly brighter in color and have stronger, possibly harsher flavors.

### 8.5. Baking Industry

**Figure 15:**
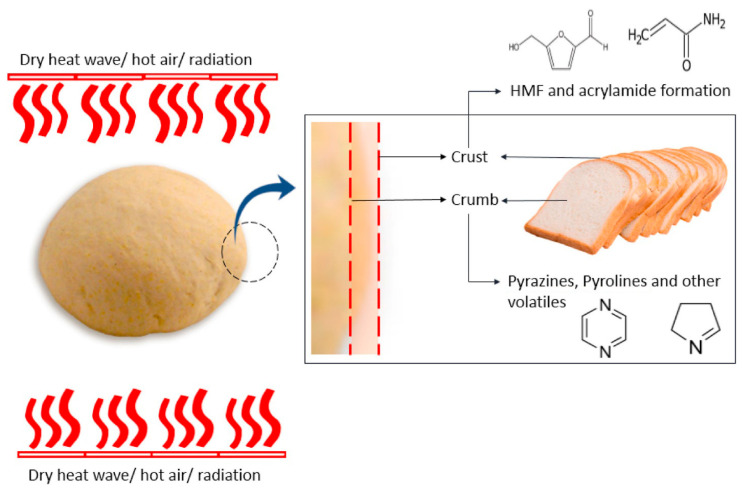
Formation of harmful compounds during baking [220].

Baking is a traditional cooking method of cereal-based products which consumers like to eat because of their sensory characteristics, such as flavor, color, texture, and aroma. The baking process involves a combination of heat and mass transfer, namely non-enzymatic browning, protein denaturation and starch gelatinization, which collectively produce the distinctive structure of the end-product [216,217]. Baked products such as bread, cookies, biscuits, crackers, cakes, brownies, and pies are very popular among consumers. Baking can be divided into three phases: dough expansion, surface desiccation, and crust browning. These phases are regulated by the temperature increase during baking, with browning typically occurring at around 160 °C. The Maillard reaction and caramelization are the two non-enzymatic browning reactions involved in the coveted color and aroma changes during baking [218]. On the other hand, during this stage of high temperature baking, toxic acrylamide and HMF can be formed, as well as numerous volatile compounds like alcohols, aldehydes, ethers, ketones, esters, acidspyrazines, pyrolines, lactones, sulfur compounds and furans. These volatile compounds are mainly present in the crumb or the crust (Figure 15) [219].

According to Zilic et al. [221], the development of MRPs in cereal-based foods is influenced by the initial levels of sugars, proteins, and total lysine. Compounds such as glyoxal (Go), methylglyoxal (MGo), dimethylglyoxal (DMGo), hydroxymethylfurfural (HMF), and 3-deoxyglucosone were found in cookies made from various cereal grains. The researchers found that Go levels were particularly high in cookies made from rye, oats, and barley. Additionally, as baking time increased, the concentration of 3-deoxyglucosone decreased, leading to a rise in HMF formation. Similar different concentrations of MRP formation were also reported by Çelik and Gokmen [222] in bread made from different cereal flours, including whole and refined wheat, rye, oats, corn, and einkorn.

## 9. Conclusions

In conclusion, the Maillard reaction is a complex chemical reaction that influences the flavor, color, and texture of many foods. Several conditions influence the reaction, including temperature, time, water activity, pH, the presence of amino acids and reducing sugars, and vitamin C. The reaction has both benefits and drawbacks in the food industry, and its nutritional repercussions are complicated and vary depending on the food matrix and the intensity of the reaction. The Maillard reaction is also important in the brewing, pharmaceutical, and cosmetic sectors. Additional research is required to better understand the impact of the Maillard reaction on health and to find techniques for decreasing the development of hazardous chemicals during food preparation and cooking.

## Figures and Tables

**Table 1:** Heterocyclic aromatic amines (HAA) that were classified by IARC as carcinogenic (2A: probably carcinogenic to humans, 2B: possibly carcinogenic to humans [130]).

Name	Abbreviation	Structure	IARC Carcinogenic Goup *
Polar HAAs (“thermic compounds”)			
2-amino-3-methylimidazo [4,5-f]quinoline	IQ	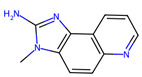	2A
2-amino-3,4-dimethylimidazo [4,5-f]quinoline	MeIQ	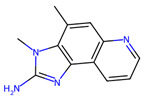	2B
2-amino-3,8-dimethylimidazo [4,5-f]quinoxaline	MeIQx	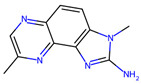	2B
2-amino-1-methyl-6- Phenylimidazo [4,5-b]pyridine	PhIP	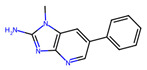	2B

* IARC classification groups: 1—carcinogenic to humans; 2A—probably carcinogenic to humans; 2B—possibly carcinogenic to humans; 3—not classifiable as to its carcinogenicity to humans.

## Data Availability

No new data were created or analyzed in this study. Data sharing is not applicable to this article.

## References

[1] Ogutu B., Kim Y.-J., Kim D.-W., Oh S.-C., Hong D.-L., Lee Y.-B. (2017). Optimization of Maillard Reaction between Glucosamine and Other Precursors by Measuring Browning with a Spectrophotometer. Prev. Nutr. Food Sci..

[2] Lund M.N., Ray C.A. (2017). Control of Maillard Reactions in Foods: Strategies and Chemical Mechanisms. J. Agric. Food Chem..

[3] Chen X., Yu J., Cui H., Xia S., Zhang X., Yang B. (2018). Effect of Temperature on Flavor Compounds and Sensory Characteristics of Maillard Reaction Products Derived from Mushroom Hydrolysate. Molecules.

[4] Tamanna N., Mahmood N. (2015). Food Processing and Maillard Reaction Products: Effect on Human Health and Nutrition. Int. J. Food Sci..

[5] Xiang J., Liu F., Wang B., Chen L., Liu W., Tan S. (2021). A Literature Review on Maillard Reaction Based on Milk Proteins and Carbohydrates in Food and Pharmaceutical Products: Advantages, Disadvantages, and Avoidance Strategies. Foods.

[6] Twarda-Clapa A., Olczak A., Białkowska A.M., Koziołkiewicz M. (2022). Advanced Glycation End-Products (AGEs): Formation, Chemistry, Classification, Receptors, and Diseases Related to AGEs. Cells.

[7] Aktağ I.G., Hamzalıoğlu A., Kocadağlı T., Gökmen V. (2022). Dietary Exposure to Acrylamide: A Critical Appraisal on the Conversion of Disregarded Intermediates into Acrylamide and Possible Reactions during Digestion. Curr. Res. Food Sci..

[8] Doseděl M., Jirkovský E., Macáková K., Krčmová L.K., Javorská L., Pourová J., Mercolini L., Remião F., Nováková L., Mladěnka P. (2021). Vitamin C—Sources, Physiological Role, Kinetics, Deficiency, Use, Toxicity, and Determination. Nutrients.

[9] Zhang L., Xia Y., Peterson D.G. (2014). Identification of Bitter Modulating Maillard-Catechin Reaction Products. J. Agric. Food Chem..

[10] Briceno Noriega D., Zenker H.E., Croes C.-A., Ewaz A., Ruinemans-Koerts J., Savelkoul H.F.J., van Neerven R.J.J., Teodorowicz M. (2022). Receptor Mediated Effects of Advanced Glycation End Products (AGEs) on Innate and Adaptative Immunity: Relevance for Food Allergy. Nutrients.

[11] Segovia Bravo K., Ramírez R., Durst R., Escobedo-Avellaneda Z.J., Welti-Chanes J., Sanz P.D., Torres J.A. (2012). Formation Risk of Toxic and Other Unwanted Compounds in Pressure-Assisted Thermally Processed Foods. J. Food Sci..

[12] ALjahdali N., Carbonero F. (2019). Impact of Maillard Reaction Products on Nutrition and Health: Current Knowledge and Need to Understand Their Fate in the Human Digestive System. Crit. Rev. Food Sci. Nutr..

[13] Charnock H.M., Pickering G.J., Kemp B.S. (2022). The Maillard Reaction in Traditional Method Sparkling Wine. Front. Microbiol..

[14] Yin X., Chen K., Cheng H., Chen X., Feng S., Song Y., Liang L. (2022). Chemical Stability of Ascorbic Acid Integrated into Commercial Products: A Review on Bioactivity and Delivery Technology. Antioxidants.

[15] Stadler R.H., Blank I., Varga N., Robert F., Hau J., Guy P.A., Robert M.-C., Riediker S. (2002). Acrylamide from Maillard Reaction Products. Nature.

[16] Jaisson S., Desmons A., Gorisse L., Gillery P. (2017). Vieillissement moléculaire des proteins—Quel rôle en physiopathologie?. Med. Sci..

[17] Peng H., Gao Y., Zeng C., Hua R., Guo Y., Wang Y., Wang Z. (2024). Effects of Maillard Reaction and Its Product AGEs on Aging and Age-Related Diseases. Food Sci. Hum. Wellness.

[18] Visvanathan R., Krishnakumar T. (2014). Acrylamide in Food Products: A Review. J. Food Process Technol..

[19] Luo Y., Li S., Ho C.-T. (2021). Key Aspects of Amadori Rearrangement Products as Future Food Additives. Molecules.

[20] Shumilina J., Kusnetsova A., Tsarev A., Janse van Rensburg H.C., Medvedev S., Demidchik V., Van den Ende W., Frolov A. (2019). Glycation of Plant Proteins: Regulatory Roles and Interplay with Sugar Signalling?. Int. J. Mol. Sci..

[21] Chen C., Zhang J.-Q., Li L., Guo M., He Y., Dong Y., Meng H., Yi F. (2022). Advanced Glycation End Products in the Skin: Molecular Mechanisms, Methods of Measurement, and Inhibitory Pathways. Front. Med..

[22] Liu S., Sun H., Ma G., Zhang T., Wang L., Pei H., Li X., Gao L. (2022). Insights into Flavor and Key Influencing Factors of Maillard Reaction Products: A Recent Update. Front. Nutr..

[23] Mertens T., Kunz T., Gibson B.R. (2022). Transition Metals in Brewing and Their Role in Wort and Beer Oxidative Stability: A Review. J. Inst. Brew..

[24] Nadkarni D.V., Sayre L.M. (1995). Structural Definition of Early Lysine and Histidine Adduction Chemistry of 4-Hydroxynonenal. Chem. Res. Toxicol..

[25] Zamora R., Alaiz M., Hidalgo F.J. (1999). Modification of Histidine Residues by 4,5-Epoxy-2-Alkenals. Chem. Res. Toxicol..

[26] Trnková L., Dršata J., Boušová I. (2015). Oxidation as an Important Factor of Protein Damage: Implications for Maillard Reaction. J. Biosci..

[27] Cincotta F., Brighina S., Condurso C., Arena E., Verzera A., Fallico B. (2021). Sugars Replacement as a Strategy to Control the Formation of α-Dicarbonyl and Furanic Compounds during Cookie Processing. Foods.

[28] Diez-Simon C., Mumm R., Hall R.D. (2019). Mass Spectrometry-Based Metabolomics of Volatiles as a New Tool for Understanding Aroma and Flavour Chemistry in Processed Food Products. Metabolomics.

[29] Govindaraju I., Sana M., Chakraborty I., Rahman M.H., Biswas R., Mazumder N. (2024). Dietary Acrylamide: A Detailed Review on Formation, Detection, Mitigation, and Its Health Impacts. Foods.

[30] Kaur N., Halford N.G. (2023). Reducing the Risk of Acrylamide and Other Processing Contaminant Formation in Wheat Products. Foods.

[31] Sharma J.K., Sihmar M., Santal A.R., Prager L., Carbonero F., Singh N.P. (2021). Barley Melanoidins: Key Dietary Compounds with Potential Health Benefits. Front. Nutr..

[32] Wang Q., Li J., Tu Y., Cai J., Ren F., Zhang H. (2023). Characteristics and Antioxidant Activity of Maillard Reaction Products from β-Lactoglobulin and Isomaltooligosaccharide. Front. Nutr..

[33] Martinez-Alvarenga M.S., Martinez-Rodriguez E.Y., Garcia-Amezquita L.E., Olivas G.I., Zamudio-Flores P.B., Acosta-Muniz C.H., Sepulveda D.R. (2014). Effect of Maillard Reaction Conditions on the Degree of Glycation and Functional Properties of Whey Protein Isolate—Maltodextrin Conjugates. Food Hydrocoll..

[34] Kutzli I., Weiss J., Gibis M. (2021). Glycation of Plant Proteins Via Maillard Reaction: Reaction Chemistry, Technofunctional Properties, and Potential Food Application. Foods.

[35] Peng Z., Zhang Y., Wang H., Gao G., Yu Z., Chong P.H., Ding W., Ke L., Zhou J., Rao P. (2021). Effects of Arginine-Glucose Maillard Reaction Products on the Physicochemical and Gel Properties of Chicken Myofibrillar Protein. LWT.

[36] Glomb M.A., Rösch D., Nagaraj R.H. (2001). Nδ-(5-Hydroxy-4,6-dimethylpyrimidine-2-yl)-l-ornithine, a Novel Methylglyoxal−Arginine Modification in Beer. J. Agric. Food Chem..

[37] Deng Y., Wierenga P.A., Schols H.A., Sforza S., Gruppen H. (2017). Effect of Maillard Induced Glycation on Protein Hydrolysis by Lysine/Arginine and Non-Lysine/Arginine Specific Proteases. Food Hydrocoll..

[38] Hwang H.-I., Hartman T.G., Ho C.-T. (1995). Relative Reactivities of Amino Acids in Pyrazine Formation. J. Agric. Food Chem..

[39] Hwang H.-I., Hartman T.G., Ho C.-T. (1995). Relative Reactivities of Amino Acids in the Formation of Pyridines, Pyrroles, and Oxazoles. J. Agric. Food Chem..

[40] Wang F., Shen H., Liu T., Yang X., Yang Y., Guo Y. (2021). Formation of Pyrazines in Maillard Model Systems: Effects of Structures of Lysine-Containing Dipeptides/Tripeptides. Foods.

[41] Biemel K.M., Reihl O., Conrad J., Lederer M.O. (2001). Formation Pathways for Lysine-Arginine Cross-Links Derived from Hexoses and Pentoses by Maillard Processes: UNRAVELING THE STRUCTURE OF A PENTOSIDINE PRECURSOR. J. Biol. Chem..

[42] Cayot P., Roullier L., Tainturier G. (1999). Electrochemical Modifications of Proteins. 1. Glycitolation. J. Agric. Food Chem..

[43] Liu P., Lu X., Li N., Zheng Z., Qiao X. (2019). Characterization, Variables, and Antioxidant Activity of the Maillard Reaction in a Fructose–Histidine Model System. Molecules.

[44] Yu H., Zhang R., Yang F., Xie Y., Guo Y., Yao W., Zhou W. (2021). Control Strategies of Pyrazines Generation from Maillard Reaction. Trends Food Sci. Technol..

[45] Nunes F.M., Del Castillo M.D., Carbonero F. (2022). Editorial: Food Melanoidins: Chemistry and Nutrition. Front. Nutr..

[46] Yang Y., Feng L., Dong X., Ma Y., Yan W., Shi X., Hu S., Yu A., Sun B. (2025). Volatile Organic Compounds Generated from the Maillard Reaction between l-Ascorbic Acid and Glycine in Hot Compressed Water. ACS Food Sci. Technol..

[47] Van Boekel M.A.J.S. (2001). Kinetic Aspects of the Maillard Reaction: A Critical Review. Food/Nahrung.

[48] Messia M.C., Caboni M.F., Marconi E. (2005). Storage Stability Assessment of Freeze-Dried Royal Jelly by Furosine Determination. J. Agric. Food Chem..

[49] Tan T.-C., Alkarkhi A.F.M., Easa A.M. (2013). Ribose-Induced Maillard Reaction as a Quality Index in Frozen Minced Chicken and Pork Meats. J. Food Qual..

[50] Akbarabadi M., Mohsenzadeh M., Housaindokht M.-R. (2020). Ribose-induced Maillard Reaction as an Analytical Method for Detection of Adulteration and Differentiation of Chilled and Frozen-thawed Minced Veal. Food Sci. Anim. Resour..

[51] Yu L., Li Q., Li Y., Yang Y., Guo C., Li M. (2021). Impact of Frozen Storage Duration of Raw Pork on the Formation of Advanced Glycation End-Products in Meatballs. LWT.

[52] Zhou Y.-Y., Li Y., Yu A.-N. (2016). The Effects of Reactants Ratios, Reaction Temperatures and Times on Maillard Reaction Products of the L-Ascorbic Acid/L-Glutamic Acid System. Food Sci. Technol..

[53] Cao J., Yan H., Liu L. (2022). Optimized Preparation and Antioxidant Activity of Glucose-Lysine Maillard Reaction Products. LWT.

[54] Feiner G., Feiner G. (2016). Chapter 3—Definitions. Salami.

[55] Liu J.-K. (2022). Natural Products in Cosmetics. Nat. Prod. Bioprospect..

[56] Kathuria D., Hamid, Gautam S., Thakur A. (2023). Maillard Reaction in Different Food Products: Effect on Product Quality, Human Health and Mitigation Strategies. Food Control.

[57] Pérez-López A.J., Noguera-Artiaga L., López-Miranda González S., Gómez-San Miguel P., Ferrández B., Carbonell-Barrachina Á.A. (2021). Acrylamide Content in French Fries Prepared with Vegetable Oils Enriched with β-Cyclodextrin or β-Cyclodextrin-Carvacrol Complexes. LWT.

[58] Seok Y.-J., Her J.-Y., Kim Y.-G., Kim M.Y., Jeong S.Y., Kim M.K., Lee J., Kim C., Yoon H.-J., Lee K.-G. (2015). Furan in Thermally Processed Foods—A Review. Toxicol. Res..

[59] Nadeem H.R., Akhtar S., Ismail T., Sestili P., Lorenzo J.M., Ranjha M.M.A.N., Jooste L., Hano C., Aadil R.M. (2021). Heterocyclic Aromatic Amines in Meat: Formation, Isolation, Risk Assessment, and Inhibitory Effect of Plant Extracts. Foods.

[60] Li Z., Zhao C., Cao C. (2023). Production and Inhibition of Acrylamide during Coffee Processing: A Literature Review. Molecules.

[61] Ren G.-R., Zhao L.-J., Sun Q., Xie H.-J., Lei Q.-F., Fang W.-J. (2015). Explore the Reaction Mechanism of the Maillard Reaction: A Density Functional Theory Study. J. Mol. Model..

[62] Koubaa M., Roohinejad S., Mungure T.E., Alaa El-Din B., Greiner R., Mallikarjunan K., Melton L., Shahidi F., Varelis P. (2019). Effect of Emerging Processing Technologies on Maillard Reactions. Encyclopedia of Food Chemistry.

[63] Nooshkam M., Varidi M., Bashash M. (2019). The Maillard Reaction Products as Food-Born Antioxidant and Antibrowning Agents in Model and Real Food Systems. Food Chem..

[64] Kchaou H., Benbettaieb N., Jridi M., Nasri M., Debeaufort F. (2019). Influence of Maillard Reaction and Temperature on Functional, Structure and Bioactive Properties of Fish Gelatin Films. Food Hydrocoll..

[65] Hedegaard R.V., Skibsted L.H., Bhandari B., Bansal N., Zhang M., Schuck P. (2013). 16—Shelf-Life of Food Powders. Handbook of Food Powders.

[66] Vera Zambrano M., Dutta B., Mercer D.G., MacLean H.L., Touchie M.F. (2019). Assessment of Moisture Content Measurement Methods of Dried Food Products in Small-Scale Operations in Developing Countries: A Review. Trends Food Sci. Technol..

[67] Wong C.W., Wijayanti H.B., Bhandari B.R., Gutiérrez-López G.F., Alamilla-Beltrán L., del Pilar Buera M., Welti-Chanes J., Parada-Arias E., Barbosa-Cánovas G.V. (2015). Maillard Reaction in Limited Moisture and Low Water Activity Environment. Water Stress in Biological, Chemical, Pharmaceutical and Food Systems.

[68] Zhang Z., Wang B., Adhikari B. (2022). Maillard Reaction between Pea Protein Isolate and Maltodextrin via Wet-Heating Route for Emulsion Stabilisation. Future Foods.

[69] Hemmler D., Roullier-Gall C., Marshall J.W., Rychlik M., Taylor A.J., Schmitt-Kopplin P. (2018). Insights into the Chemistry of Non-Enzymatic Browning Reactions in Different Ribose-Amino Acid Model Systems. Sci. Rep..

[70] Mehta B.M., Cheung P.C.K., Mehta B.M. (2014). Nutritional and Toxicological Aspects of the Chemical Changes of Food Components and Nutrients During Heating and Cooking. Handbook of Food Chemistry.

[71] Ephrem E., Najjar A., Charcosset C., Greige-Gerges H. (2018). Encapsulation of Natural Active Compounds, Enzymes, and Probiotics for Fruit Juice Fortification, Preservation, and Pro-cessing: An Overview. J. Funct. Foods.

[72] Sogut E., Ertekin Filiz B., Seydim A.C. (2021). A Model System Based on Glucose–Arginine to Monitor the Properties of Maillard Reaction Products. J. Food Sci. Technol..

[73] Somjai C., Siriwoharn T., Kulprachakarn K., Chaipoot S., Phongphisutthinant R., Wiriyacharee P. (2021). Utilization of Maillard Reaction in Moist-Dry-Heating System to Enhance Physicochemical and Antioxidative Properties of Dried Whole Longan Fruit. Heliyon.

[74] Akıllıoğlu H.G., Chatterton D.E.W., Lund M.N. (2022). Maillard Reaction Products and Amino Acid Cross-Links in Liquid Infant Formula: Effects of UHT Treatment and Storage. Food Chem..

[75] Wang Y., Hu H., McClements D.J., Nie S., Shen M., Li C., Huang Y., Chen J., Zeng M., Xie M. (2019). Effect of Fatty Acids and Triglycerides on the Formation of Lysine-Derived Advanced Glycation End-Products in Model Systems Exposed to Frying Temperature. RSC Adv..

[76] Tongtummachat T., Akkarawatkhoosith N., Kaewchada A., Jaree A. (2020). Conversion of Glucose to 5-Hydroxymethylfurfural in a Microreactor. Front. Chem..

[77] Shahidi F., Hossain A. (2022). Role of Lipids in Food Flavor Generation. Molecules.

[78] Wan C., Wang Y., Lian C., Chang Q., An Y., Chen J., Sun J., Hou Z., Yang D., Guo X. (2022). Histidine-Specific Bioconjugation via Visible-Light-Promoted Thioacetal Activation. Chem. Sci..

[79] Pogostin B.H., Malmendal A., Londergan C.H., Åkerfeldt K.S. (2019). pKa Determination of a Histidine Residue in a Short Peptide Using Raman Spectroscopy. Molecules.

[80] Fitch C.A., Platzer G., Okon M., Garcia-Moreno E. B., McIntosh L.P. (2015). Arginine: Its pKa Value Revisited. Protein Sci..

[81] Harms M.J., Schlessman J.L., Sue G.R., García-Moreno E. B. (2011). Arginine Residues at Internal Positions in a Protein Are Always Charged. Proc. Natl. Acad. Sci. USA.

[82] Thorpe S.R., Baynes J.W. (2003). Maillard Reaction Products in Tissue Proteins: New Products and New Perspectives. Amino Acids.

[83] Zha F., Gao K., Rao J., Chen B. (2021). Maillard-Driven Chemistry to Tune the Functionality of Pea Protein: Structure Characterization, Site-Specificity, and Aro-matic Profile. Trends Food Sci. Technol..

[84] Zhang M., Vogel H.J. (1993). etermination of the Side Chain pKa Values of the Lysine Residues in Calmodulin. J. Biol. Chem..

[85] Isom D.G., Castañeda C.A., Cannon B.R., García-Moreno E. B. (2011). Large Shifts in pKa Values of Lysine Residues Buried inside a Protein. Proc. Natl. Acad. Sci. USA.

[86] Dyer J.M., Clerens S., Grosvenor A., Thomas A., Callaghan C., Deb-Choudhury S., Haines S. (2016). Proteomic Tracking of Hydrothermal Maillard and Redox Modification in Lactoferrin and β-Lactoglobulin: Location of Lactosylation, Carboxymethylation, and Oxidation Sites. J. Dairy. Sci..

[87] Meltretter J., Seeber S., Humeny A., Becker C.-M., Pischetsrieder M. (2007). Site-Specific Formation of Maillard, Oxidation, and Condensation Products from Whey Proteins during Reaction with Lactose. J. Agric. Food Chem..

[88] Cayot P., Lorient D. (1997). Structures et Technofonctions des Protéines du Lait—Philippe CAYOT, Denis Lorient (EAN13: 9782743017460)|e.lavoisier—Ma Librairie Ebooks.

[89] Wieser H. (2007). Chemistry of Gluten Proteins. Food Microbiol..

[90] Calhoun W.K., Hepburn F.N., Bradley W.B. (1960). The Availability of Lysine in Wheat, Flour, Bread and Gluten. J. Nutr..

[91] Woychik J.H., Boundy J.A., Dimler R.J. (1961). Wheat Gluten Proteins, Amino Acid Composition of Proteins in Wheat Gluten. J. Agric. Food Chem..

[92] Chen T., Wei C.-K., Li T., Zhang H.-L., Ni Z.-J., Khan M.R., Wei Z.-J. (2023). Effects of Reducing Sugars on the Structural and Flavor Properties of the Maillard Reaction Products of Lycium Barbarum Seed Meal. Foods.

[93] Lin H.-T.V., Chan D.-S., Kao L.-Y., Sung W.-C. (2021). Effect of Hydroxymethylfurfural and Low-Molecular-Weight Chitosan on Formation of Acrylamide and Hydroxymethylfurfural during Maillard Reaction in Glucose and Asparagine Model Systems. Polymers.

[94] Belitz H.-D., Grosch W., Schieberle P. (2009). Springer Food Chemistry.

[95] Han Z., Gao J., Wang X., Wang W., Dong J., Zhang Y., Wang S. (2019). Formation and Alterations of the Potentially Harmful Maillard Reaction Products during the Production and Storage of Brown Fermented Milk. Molecules.

[96] Moldoveanu S.C. (2021). Analytical Pyrolysis of Caramel Colours and of Maillard Browning Polymers. Anal. Pyrolysis Nat. Org. Polym..

[97] Laemont J., Barringer S. (2023). Effect of pH, Reducing Sugars, and Protein on Roasted Sunflower Seed Aroma Volatiles. Foods.

[98] Smuda M., Glomb M.A. (2013). Maillard Degradation Pathways of Vitamin C. Angew. Chem. Int. Ed..

[99] Zhong C., Tan S., Langrish T. (2019). Redness Generation via Maillard Reactions of Whey Protein Isolate (WPI) and Ascorbic Acid (Vitamin C) in Spray-Dried Powders. J. Food Eng..

[100] Rufián-Henares J.A., Pastoriza S., Caballero B., Finglas P.M., Toldrá F. (2016). Maillard Reaction. Encyclopedia of Food and Health.

[101] Augustine D.A., Bent G.-A. (2022). Acrylamide, a Toxic Maillard by-Product and Its Inhibition by Sulfur-Containing Compounds: A Mini Review. Front. Food. Sci. Technol..

[102] Awoonor-Williams E., Rowley C.N. (2016). Evaluation of Methods for the Calculation of the pKa of Cysteine Residues in Proteins. J. Chem. Theory Comput..

[103] Kallis G.B., Holmgren A. (1980). Differential Reactivity of the Functional Sulfhydryl Groups of Cysteine-32 and Cysteine-35 Present in the Reduced Form of Thioredoxin from Escherichia Coli. J. Biol. Chem..

[104] Kortemme T., Creighton T.E. (1995). Ionisation of Cysteine Residues at the Termini of Model α-Helical Peptides. Relevance to Unusual Thiol p*K*aValues in Proteins of the Thioredoxin Family. J. Mol. Biol..

[105] Kella N.K.D., Kinsella J.E. (1988). Structural Stability of β-Lactoglobulin in the Presence of Kosmotropic Salts A Kinetic and Thermodynamic Study. Int. J. Pept. Protein Res..

[106] Lienhard G.E., Jencks W.P. (1966). Thiol Addition to the Carbonyl Group. Equilibria and Kinetics1. J. Am. Chem. Soc..

[107] Yang S., Zhang Z., Li J., Niu Y., Yu L.L. (2021). Inhibition Mechanism of L-Cysteine on Maillard Reaction by Trapping 5-Hydroxymethylfurfural. Foods.

[108] Nielsen S.D., Knudsen L.J., Bækgaard L.T., Rauh V., Larsen L.B. (2022). Influence of Lactose on the Maillard Reaction and Dehydroalanine-Mediated Protein Cross-Linking in Casein and Whey. Foods.

[109] Nisbet A.D., Saundry R.H., Moir A.J.G., Fothergill L.A., Fothergill J.E. (1981). The Complete Amino-Acid Sequence of Hen Ovalbumin. Eur. J. Biochem..

[110] Campanella B., Onor M., D’Ulivo A., Giannarelli S., Bramanti E. (2014). Impact of Protein Concentration on the Determination of Thiolic Groups of Ovalbumin: A Size Exclusion Chromatography–Chemical Vapor Generation–Atomic Fluorescence Spectrometry Study via Mercury Labeling. Anal. Chem..

[111] Noda K., Terasawa N., Murata M. (2016). Formation Scheme and Antioxidant Activity of a Novel Maillard Pigment, Pyrrolothiazolate, Formed from Cysteine and Glucose. Food Funct..

[112] Sun Y., Zhang P., Wang X., Al-Zahrani F.A.M., de Leeuw N.H., Lin L. (2022). Deciphering Key Coloured Compounds from Sunless Tanning Reactions. Dye. Pigment..

[113] Zhang Z., He S., Zhang L., Li X., Jin R., Liu Q., Chen S., Wang J., Sun H. (2022). The Potential Application of Vegetable Oils in the D-Xylose and L-Cysteine Maillard Reaction System for Meaty Aroma Production. Food Res. Int..

[114] Yu A.-N., Tan Z.-W., Wang F.-S. (2012). Mechanism of Formation of Sulphur Aroma Compounds from L-Ascorbic Acid and l-Cysteine during the Maillard Reaction. Food Chem..

[115] Billaud C., Maraschin C., Peyrat-Maillard M.-N., Nicolas J. (2005). Maillard Reaction Products Derived from Thiol Compounds as Inhibitors of Enzymatic Browning of Fruits and Vegetables: The Structure-Activity Relationship. Ann. N. Y. Acad. Sci..

[116] Hofmann T., Schieberle P. (2002). Chemical Interactions between Odor-Active Thiols and Melanoidins Involved in the Aroma Staling of Coffee Beverages. J. Agric. Food Chem..

[117] Kukuminato S., Koyama K., Koseki S. (2021). Antibacterial Properties of Melanoidins Produced from Various Combinations of Maillard Reaction against Pathogenic Bacteria. Microbiol. Spectr..

[118] Karanth S., Feng S., Patra D., Pradhan A.K. (2023). Linking Microbial Contamination to Food Spoilage and Food Waste: The Role of Smart Packaging, Spoilage Risk Assess-ments, and Date Labeling. Front. Microbiol..

[119] Quinto E.J., Caro I., Villalobos-Delgado L.H., Mateo J., De-Mateo-Silleras B., Redondo-Del-Río M.P. (2019). Food Safety through Natural Antimicrobials. Antibiotics.

[120] Wen F., Zeng C., Yang Y., Xu T., Wang H., Wang S. (2023). Sensory Attributes and Functional Properties of Maillard Reaction Products Derived from the Crassosotrea Gigas (Ostrea Rivularis Gould) Enzymatic Hydrolysate and Xylose System. Heliyon.

[121] Bolchini S., Angeli L., Ferrentino G., Van Boekel M.A.J.S., Amorati R., Scampicchio M., Morozova K. (2025). Free Radical Scavenging Kinetics of Maillard Reaction Products: A Glucose-Glycine Model System. LWT.

[122] Pruteanu L.L., Bailey D.S., Grădinaru A.C., Jäntschi L. (2023). The Biochemistry and Effectiveness of Antioxidants in Food, Fruits, and Marine Algae. Antioxidants.

[123] Yu J., Hu N., Hou L., Hang F., Li K., Xie C. (2023). Extraction Methods of Melanoidins and Its Potential as a Natural Pigment. Food Sci. Technol..

[124] Martinez-Gomez A., Caballero I., Blanco C.A. (2020). Phenols and Melanoidins as Natural Antioxidants in Beer. Structure, Reactivity and Antioxidant Activity. Biomolecules.

[125] Etcheverry P., Grusak M.A., Fleige L.E. (2012). Application of in Vitro Bioaccessibility and Bioavailability Methods for Calcium, Carotenoids, Folate, Iron, Magnesium, Polyphenols, Zinc, and Vitamins B6, B12, D, and E. Front. Physiol..

[126] Fanelli N.S., Bailey H.M., Guardiola L.V., Stein H.H. (2021). Values for Digestible Indispensable Amino Acid Score (DIAAS) Determined in Pigs Are Greater for Milk Than for Breakfast Cereals, but DIAAS Values for Individual Ingredients Are Additive in Combined Meals. J. Nutr..

[127] Fanelli N.S., Bailey H.M., Thompson T.W., Delmore R., Nair M.N., Stein H.H. (2022). Digestible Indispensable Amino Acid Score (DIAAS) Is Greater in Animal-Based Burgers than in Plant-Based Burgers If De-termined in Pigs. Eur. J. Nutr..

[128] Ajomiwe N., Boland M., Phongthai S., Bagiyal M., Singh J., Kaur L. (2024). Protein Nutrition: Understanding Structure, Digestibility, and Bioavailability for Optimal Health. Foods.

[129] Gül Akıllıoğlu H., Savaş Bahçeci K., Gökmen V. (2015). Investigation and Kinetic Evaluation of Furan Formation in Tomato Paste and Pulp during Heating. Food Res. Int..

[130] Bulanda S., Janoszka B. (2022). Consumption of Thermally Processed Meat Containing Carcinogenic Compounds (Polycyclic Aromatic Hydrocarbons and Heterocyclic Aromatic Amines) versus a Risk of Some Cancers in Humans and the Possibility of Reducing Their Formation by Natural Food Additives—A Literature Review. Int. J. Environ. Res. Public Health.

[131] Delgado-Andrade C. (2016). Carboxymethyl-Lysine: Thirty Years of Investigation in the Field of AGE Formation. Food Funct..

[132] Zhang Y., Ren Y., Zhang Y. (2009). New Research Developments on Acrylamide: Analytical Chemistry, Formation Mechanism, and Mitigation Recipes. Chem. Rev..

[133] Yan F., Wang L., Zhao L., Wang C., Lu Q., Liu R. (2023). Acrylamide in Food: Occurrence, Metabolism, Molecular Toxicity Mechanism and Detoxification by Phytochemicals. Food Chem. Toxicol..

[134] EFSA Panel on Contaminants in the Food (2015). Scientific Opinion on Acrylamide in Food. EFSA J..

[135] Koszucka A., Nowak A. (2019). Thermal Processing Food-Related Toxicants: A Review. Crit. Rev. Food Sci. Nutr..

[136] Knutsen H.K., Alexander J., Barregård L., Bignami M., Brüschweiler B., Ceccatelli S., Cottrill B., Dinovi M., Edler L., EFSA Panel on Contaminants in the Food (2017). Risks for Public Health Related to the Presence of Furan and Methylfurans in Food. EFSA J..

[137] Friedman M. (2003). Chemistry, Biochemistry, and Safety of Acrylamide. A Review. J. Agric. Food Chem..

[138] Barzegar F., Kamankesh M., Mohammadi A. (2023). Recent Development in Formation, Toxic Effects, Human Health and Analytical Techniques of Food Contaminants. Food Rev. Int..

[139] Gironés-Vilaplana A., Villaño D., Marhuenda J., Moreno D.A., García-Viguera C., Galanakis C.M. (2017). Chapter 6—Vitamins. Nutraceutical and Functional Food Components.

[140] Murata M. (2021). Browning and Pigmentation in Food through the Maillard Reaction. Glycoconj. J..

[141] Mohd Hashim M.N., Abd-Talib N., Yaji E.L.A., Kelly Y.T.L., Razali N., Pa’ee K.F. (2021). The Effect of Frying on Browning, Acrylamide and 5-Hydroxymethylfurfural Formation on Malaysian Curry Puff Skin Treated with l-Asparaginase. Food Sci. Biotechnol..

[142] Qiu J., Li H., Liu Y., Li C., Fang Z., Hu B., Li X., Zeng Z., Liu Y. (2024). Changes in Flavor and Biological Activities of Lentinula Edodes Hydrolysates after Maillard Reaction. Food Chem..

[143] Nooshkam M., Varidi M., Litwack G. (2024). Chapter Twelve—Antioxidant and Antibrowning Properties of Maillard Reaction Products in Food and Biological Systems. Vitamins and Hormones.

[144] Tran T.N., Doan C.T., Nguyen V.B., Nguyen A.D., Wang S.-L. (2019). Anti-Oxidant and Anti-Diabetes Potential of Water-Soluble Chitosan–Glucose Derivatives Produced by Maillard Reaction. Polymers.

[145] Hwang I.G., Kim H.Y., Woo K.S., Lee J., Jeong H.S. (2011). Biological Activities of Maillard Reaction Products (MRPs) in a Sugar–Amino Acid Model System. Food Chem..

[146] Kitts D.D., Chen X.-M., Jing H. (2012). Demonstration of Antioxidant and Anti-Inflammatory Bioactivities from Sugar–Amino Acid Maillard Reaction Products. J. Agric. Food Chem..

[147] Seiquer I., Rubio L.A., Peinado M.J., Delgado-Andrade C., Navarro M.P. (2014). Maillard Reaction Products Modulate Gut Microbiota Composition in Adolescents. Mol. Nutr. Food Res..

[148] Liang Y., Zhang H., Tian L., Shi C., Zheng Y., Wang J., Tan Y., Luo Y., Hong H. (2023). Gut Microbiota and Metabolic Profile as Affected by Maillard Reaction Products Derived from Bighead Carp Meat Hydrol-ysates with Galactose and Galacto-Oligosaccharides during in Vitro Pig Fecal Fermentation. Food Chem..

[149] Gupta R.K., Kriti G., Akanksha S., Mukul D., Irfan A., Dwivedi P.D. (2018). Maillard Reaction in Food Allergy: Pros and Cons. Crit. Rev. Food Sci. Nutr..

[150] Kong S.Y., Takeuchi M., Hyogo H., McKeown-Eyssen G., Yamagishi S., Chayama K., O’Brien P.J., Ferrari P., Overvad K., Olsen A. (2015). The Association between Glyceraldehyde-Derived Advanced Glycation End-Products and Colorectal Cancer Risk. Cancer Epidemiol. Biomark. Prev..

[151] Monnier V.M., Taniguchi N. (2016). Advanced Glycation in Diabetes, Aging and Age-Related Diseases: Editorial and Dedication. Glycoconj. J..

[152] Vicente Miranda H., Szegő É.M., Oliveira L.M.A., Breda C., Darendelioglu E., de Oliveira R.M., Ferreira D.G., Gomes M.A., Rott R., Oliveira M. (2017). Glycation Potentiates α-Synuclein-Associated Neurodegeneration in Synucleinopathies. Brain.

[153] Zhang Y., Huang M., Wang Q., Cheng J. (2016). Structure-Guided Unravelling: Phenolic Hydroxyls Contribute to Reduction of Acrylamide Using Multiplex Quantitative Structure–Activity Relationship Modelling. Food Chem..

[154] Constantinou C., Koutsidis G. (2016). Investigations on the Effect of Antioxidant Type and Concentration and Model System Matrix on Acrylamide Formation in Model Maillard Reaction Systems. Food Chem..

[155] Bhuiyan M.N.I., Mitsuhashi S., Sigetomi K., Ubukata M. (2017). Quercetin Inhibits Advanced Glycation End Product Formation via Chelating Metal Ions, Trapping Methylglyoxal, and Trapping Reactive Oxygen Species. Biosci. Biotechnol. Biochem..

[156] Culetu A., Fernandez-Gomez B., Ullate M., del Castillo M.D., Andlauer W. (2016). Effect of Theanine and Polyphenols Enriched Fractions from Decaffeinated Tea Dust on the Formation of Maillard Reaction Products and Sensory Attributes of Breads. Food Chem..

[157] Shen Y., Xu Z., Sheng Z. (2017). Ability of Resveratrol to Inhibit Advanced Glycation End Product Formation and Carbohydrate-Hydrolyzing Enzyme Ac-tivity, and to Conjugate Methylglyoxal. Food Chem..

[158] Zhao T., Xi J., Zhang C., Ma Y., Wang X. (2021). Using Adinandra Nitida Leaf Extract to Prevent Heterocyclic Amine Formation in Fried Chicken Patties. RSC Adv..

[159] Fu Y., Jia Y., Sun Y., Liu X., Yi J., Cai S. (2022). Dietary Flavonoids Alleviate Inflammation and Vascular Endothelial Barrier Dysfunction Induced by Advanced Glycation End Products In Vitro. Nutrients.

[160] Hazra S., Hossain M., Kumar G.S. (2014). Studies on α-, β-, and γ-Cyclodextrin Inclusion Complexes of Isoquinoline Alkaloids Berberine, Palmatine and Coralyne. J. Incl. Phenom. Macrocycl. Chem..

[161] Tang M., Cheng L., Liu Y., Wu Z., Zhang X., Luo S. (2022). Plant Polysaccharides Modulate Immune Function via the Gut Microbiome and May Have Potential in COVID-19 Therapy. Molecules.

[162] Zhu R., Hong M., Zhuang C., Zhang L., Wang C., Liu J., Duan Z., Shang F., Hu F., Li T. (2019). Pectin Oligosaccharides from Hawthorn (Crataegus Pinnatifida Bunge. Var. Major) Inhibit the Formation of Advanced Gly-cation End Products in Infant Formula Milk Powder. Food Funct..

[163] Masyita A., Mustika Sari R., Dwi Astuti A., Yasir B., Rahma Rumata N., Emran T.B., Nainu F., Simal-Gandara J. (2022). Terpenes and Terpenoids as Main Bioactive Compounds of Essential Oils, Their Roles in Human Health and Potential Ap-plication as Natural Food Preservatives. Food Chem. X.

[164] Parvin R., Seo J., Eom J.-U., Ahamed Z., Yang H.-S. (2023). Inhibitory and Antioxidative Capacity of Nutmeg Extracts on Reduction of Lipid Oxidation and Heterocyclic Amines in Pan-Roasted Beef Patties. Meat Sci..

[165] Gumus D., Kizil M. (2022). Comparison of the Reducing Effects of Blueberry and Propolis Extracts on Heterocyclic Aromatic Amines Formation in Pan Fried Beef. Meat Sci..

[166] Jing Y., Li X., Hu X., Ma Z., Liu L., Ma X. (2019). Effect of Buckwheat Extracts on Acrylamide Formation and the Quality of Bread. J. Sci. Food Agric..

[167] Teng H., Chen Y., Lin X., Lv Q., Chai T.-T., Wong F.-C., Chen L., Xiao J. (2019). Inhibitory Effect of the Extract from Sonchus Olearleu on the Formation of Carcinogenic Heterocyclic Aromatic Amines dur-ing the Pork Cooking. Food Chem. Toxicol..

[168] Macit A., Kizil M. (2022). Effect of Olive Leaf Extract Marination on Heterocyclic Aromatic Amine Formation in Pan-Fried Salmon. J. Sci. Food Agric..

[169] Trujillo-Mayol I., Madalena C., Sobral M., Viegas O., Cunha S.C., Alarcón-Enos J., Pinho O., Ferreira I.M.P.L.V.O. (2021). Incorporation of Avocado Peel Extract to Reduce Cooking-Induced Hazards in Beef and Soy Burgers: A Clean Label Ingre-dient. Food Res. Int..

[170] Tengilimoglu-Metin M.M., Hamzalioglu A., Gokmen V., Kizil M. (2017). Inhibitory Effect of Hawthorn Extract on Heterocyclic Aromatic Amine Formation in Beef and Chicken Breast Meat. Food Res. Int..

[171] Khan I.A., Liu D., Yao M., Memon A., Huang J., Huang M. (2019). Inhibitory Effect of Chrysanthemum Morifolium Flower Extract on the Formation of Heterocyclic Amines in Goat Meat Patties Cooked by Various Cooking Methods and Temperatures. Meat Sci..

[172] Khan M.R., Busquets R., Azam M. (2021). Blueberry, Raspberry, and Strawberry Extracts Reduce the Formation of Carcinogenic Heterocyclic Amines in Fried Camel, Beef and Chicken Meats. Food Control.

[173] Li M., Lin S., Wang R., Gao D., Bao Z., Chen D., Tang Y., Sun N., Zhang S. (2022). Inhibitory Effect and Mechanism of Various Fruit Extracts on the Formation of Heterocyclic Aromatic Amines and Flavor Changes in Roast Large Yellow Croaker (*Pseudosciaena crocea*). Food Control.

[174] Zhou Y., Zhang M., Ma Z., Li Z., Ma Q., Wang L. (2024). Effect of Spices on the Formation and Inhibition of Heterocyclic Amines in Barbecued Pork. Food Meas..

[175] Yang D., He Z., Wang Z., Fang Q., Oz F., Chen J., Zeng M. (2022). Processing Stage-Guided Effects of Spices on the Formation and Accumulation of Heterocyclic Amines in Smoked and Cooked Sausages. Food Biosci..

[176] Arámbula-Villa G., Flores-Casamayor V., Velés-Medina J.J., Salazar R. (2018). Mitigating Effect of Calcium and Magnesium on Acrylamide Formation in Tortilla Chips. Cereal Chem..

[177] Zhu Y., Luo Y., Sun G., Wang P., Hu X., Chen F. (2020). Role of Glutathione on Acrylamide Inhibition: Transformation Products and Mechanism. Food Chem..

[178] Qi Y., Zhang H., Wu G., Zhang H., Gu L., Wang L., Qian H., Qi X. (2018). Mitigation Effects of Proanthocyanidins with Different Structures on Acrylamide Formation in Chemical and Fried Potato Crisp Models. Food Chem..

[179] Qi Y., Zhang H., Wu G., Zhang H., Wang L., Qian H., Qi X. (2018). Reduction of 5-Hydroxymethylfurfural Formation by Flavan-3-Ols in Maillard Reaction Models and Fried Potato Chips. J. Sci. Food Agric..

[180] Heydari Ashkezari M., Salehifar M. (2019). Inhibitory Effects of Pomegranate Flower Extract and Vitamin B3 on the Formation of Acrylamide during the Donut Making Process. Food Meas..

[181] Yang H., Li L., Yin Y., Li B., Zhang X., Jiao W., Liang Y. (2019). Effect of Ground Ginger on Dough and Biscuit Characteristics and Acrylamide Content. Food Sci. Biotechnol..

[182] Zhu Y., Luo Y., Sun G., Wang P., Hu X., Chen F. (2022). The Simultaneous Inhibition of Histidine on 5-Hydroxymethylfurfural and Acrylamide in Model Systems and Cookies. Food Chem..

[183] Pedreschi F., Saavedra I., Bunger A., Zuñiga R.N., Pedreschi R., Chirinos R., Campos D., Mariotti-Celis M.S. (2018). Tara Pod (Caesalpinia Spinosa) Extract Mitigates Neo-Contaminant Formation in Chilean Bread Preserving Their Sensory Attributes. LWT.

[184] Zhang Y., An X. (2017). Inhibitory Mechanism of Quercetin against the Formation of 5-(Hydroxymethyl)-2-Furaldehyde in Buckwheat Flour Bread by Ultra-Performance Liquid Chromatography Coupled with High-Resolution Tandem Mass Spectrometry. Food Res. Int..

[185] Abrantes T., Moura-Nunes N., Perrone D. (2022). Gallic Acid Mitigates 5-Hydroxymethylfurfural Formation While Enhancing or Preserving Browning and Antioxidant Activity Development in Glucose/Arginine and Sucrose/Arginine Maillard Model Systems. Molecules.

[186] Khaneghah A.M., Gavahian M., Xia Q., Denoya G.I., Roselló-Soto E., Barba F.J., Barba F.J., Parniakov O., Wiktor A. (2020). 6—Effect of Pulsed Electric Field on Maillard Reaction and Hydroxymethylfurfural Production. Pulsed Electric Fields to Obtain Healthier and Sustainable Food for Tomorrow.

[187] Michalak J., Czarnowska-Kujawska M., Klepacka J., Gujska E. (2020). Effect of Microwave Heating on the Acrylamide Formation in Foods. Molecules.

[188] Sansano M., De los Reyes R., Andrés A., Heredia A. (2018). Effect of Microwave Frying on Acrylamide Generation, Mass Transfer, Color, and Texture in French Fries. Food Bioprocess. Technol..

[189] Tsevdou M., Ntzimani A., Katsouli M., Dimopoulos G., Tsimogiannis D., Taoukis P. (2024). Comparative Study of Microwave, Pulsed Electric Fields, and High Pressure Processing on the Extraction of Antioxidants from Olive Pomace. Molecules.

[190] Yi J., Kebede B.T., Hai Dang D.N., Buvé C., Grauwet T., Van Loey A., Hu X., Hendrickx M. (2017). Quality Change during High Pressure Processing and Thermal Processing of Cloudy Apple Juice. LWT.

[191] Avila Ruiz G., Xi B., Minor M., Sala G., van Boekel M., Fogliano V., Stieger M. (2016). High-Pressure–High-Temperature Processing Reduces Maillard Reaction and Viscosity in Whey Protein–Sugar Solutions. J. Agric. Food Chem..

[192] Cappato L.P., Ferreira M.V.S., Guimaraes J.T., Portela J.B., Costa A.L.R., Freitas M.Q., Cunha R.L., Oliveira C.A.F., Mercali G.D., Marzack L.D.F. (2017). Ohmic Heating in Dairy Processing: Relevant Aspects for Safety and Quality. Trends Food Sci. Technol..

[193] Silva R., Rocha R.S., Guimarães J.T., Balthazar C.F., Scudino H., Ramos G.L.P.A., Pimentel T.C., Silva M.C., Henrique F., Silva P. (2020). Dulce de Leche Submitted to Ohmic Heating Treatment: Consumer Sensory Profile Using Preferred Attribute Elicitation (PAE) and Temporal Check-All-That-Apply (TCATA). Food Res. Int..

[194] Pires R.P.S., Cappato L.P., Guimarães J.T., Rocha R.S., Silva R., Balthazar C.F., Freitas M.Q., Silva P.H.F., Neto R.P.C., Tavares M.I.B. (2020). Ohmic Heating for Infant Formula Processing: Evaluating the Effect of Different Voltage Gradient. J. Food Eng..

[195] Zhang X., Zhang M., Adhikari B. (2020). Recent Developments in Frying Technologies Applied to Fresh Foods. Trends Food Sci. Technol..

[196] Verma V., Singh V., Chauhan O.P., Yadav N. (2023). Comparative Evaluation of Conventional and Advanced Frying Methods on Hydroxymethylfurfural and Acrylamide For-mation in French Fries. Innov. Food Sci. Emerg. Technol..

[197] Belkova B., Hradecky J., Hurkova K., Forstova V., Vaclavik L., Hajslova J. (2018). Impact of Vacuum Frying on Quality of Potato Crisps and Frying Oil. Food Chem..

[198] Devseren E., Okut D., Koç M., Ocak Ö.Ö., Karataş H., Kaymak-Ertekin F. (2021). Effect of Vacuum Frying Conditions on Quality of French Fries and Frying Oil. Acta Chim. Slov..

[199] Luo D., Pan X., Zhang W., Bi S., Wu J. (2022). Effect of Glucose Oxidase Treatment on the Aroma Qualities and Release of Cooked Off-Odor Components from Heat-Treated Hami Melon Juice. Food Chem..

[200] Alam S., Ahmad R., Pranaw K., Mishra P., Khare S.K. (2018). Asparaginase Conjugated Magnetic Nanoparticles Used for Reducing Acrylamide Formation in Food Model System. Bioresour. Technol..

[201] Xu F., Oruna-Concha M.-J., Elmore J.S. (2016). The Use of Asparaginase to Reduce Acrylamide Levels in Cooked Food. Food Chem..

[202] Lemos A.C., de Borba V.S., Burkert J.F.d.M., Scaglioni P.T., Badiale-Furlong E. (2023). Role of White Bread Matrix Components and Processing Parameters on 5-Hydroxymethylfurfural (HMF) and Acrylamide Formation. Food Control.

[203] Shen Y., Chen G., Li Y. (2018). Bread Characteristics and Antioxidant Activities of Maillard Reaction Products of White Pan Bread Containing Various Sug-ars. LWT.

[204] Parisi S., Luo W., Parisi S., Luo W. (2018). The Importance of Maillard Reaction in Processed Foods. Chemistry of Maillard Reactions in Processed Foods.

[205] Hosen A., Al-Mamun A., Robin M.A., Habiba U., Sultana R. (2021). Maillard Reaction: Food Processing Aspects. North Am. Acad. Res..

[206] Kim I.-S., Kim C.-H., Yang W.-S. (2021). Physiologically Active Molecules and Functional Properties of Soybeans in Human Health—A Current Perspective. Int. J. Mol. Sci..

[207] Kubo M.T.K., dos Reis B.H.G., Sato L.N.I., Gut J.A.W. (2021). Microwave and Conventional Thermal Processing of Soymilk: Inactivation Kinetics of Lipoxygenase and Trypsin Inhibitors Activity. LWT.

[208] Kowalski S., Lukasiewicz M., Duda-Chodak A., Zięć G. (2013). 5-Hydroxymethyl-2-Furfural (HMF)—Heat-Induced Formation, Occurrence in Food and Biotransformation—A Review. Pol. J. Food Nutr. Sci..

[209] Krishna T.C., Najda A., Bains A., Tosif M.M., Papliński R., Kapłan M., Chawla P. (2021). Influence of Ultra-Heat Treatment on Properties of Milk Proteins. Polymers.

[210] Goulding D.A., Fox P.F., O’Mahony J.A., Boland M., Singh H. (2020). Chapter 2—Milk Proteins: An Overview. Milk Proteins.

[211] Li L., Belloch C., Flores M. (2021). The Maillard Reaction as Source of Meat Flavor Compounds in Dry Cured Meat Model Systems under Mild Temperature Conditions. Molecules.

[212] Dong Z.Y., Liu W., Zhou Y.J., Ren H., Li M.Y., Liu Y. (2019). Effects of Ultrasonic Treatment on Maillard Reaction and Product Characteristics of Enzymatic Hydrolysate Derived from Mussel Meat. J. Food Process Eng..

[213] Semedo Tavares W.P., Dong S., Jin W., Yang Y., Han K., Zha F., Zhao Y., Zeng M. (2018). Effect of Different Cooking Conditions on the Profiles of Maillard Reaction Products and Nutrient Composition of Hairtail (Thichiurus Lepturus) Fillets. Food Res. Int..

[214] Yıldırım H.K., Ray R.C. (2021). 2—Insights into the Role of Yeasts in Alcoholic Beverages. Microbial Biotechnology in Food and Health.

[215] Baxter E.D., Hughes P.S. (2001). Beer: Quality, Safety and Nutritional Aspects.

[216] Nematollahi A., Mollakhalili Meybodi N., Mousavi Khaneghah A. (2021). An Overview of the Combination of Emerging Technologies with Conventional Methods to Reduce Acrylamide in Different Food Products: Perspectives and Future Challenges. Food Control.

[217] Sruthi N.U., Premjit Y., Pandiselvam R., Kothakota A., Ramesh S.V. (2021). An Overview of Conventional and Emerging Techniques of Roasting: Effect on Food Bioactive Signatures. Food Chem..

[218] Pico J., Bernal J., Gómez M. (2015). Wheat Bread Aroma Compounds in Crumb and Crust: A Review. Food Res. Int..

[219] Nguyen H.T., Van der Fels-Klerx H.J., Peters R.J.B., Van Boekel M.A.J.S. (2016). Acrylamide and 5-Hydroxymethylfurfural Formation during Baking of Biscuits: Part I: Effects of Sugar Type. Food Chem..

[220] Devu S.S., Dileepmon R., Kothakota A., Venkatesh T., Pandiselvam R., Garg R., Jambrak A., Mediboyina M.K., Kumar M., Rajkumar (2022). Recent Advancements in Baking Technologies to Mitigate Formation of Toxic Compounds: A Comprehensive Review. Food Control.

[221] Žilić S., Aktağ I.G., Dodig D., Gökmen V. (2021). Investigations on the Formation of Maillard Reaction Products in Sweet Cookies Made of Different Cereals. Food Res. Int..

[222] Çelik E.E., Gökmen V. (2020). Formation of Maillard Reaction Products in Bread Crust-like Model System Made of Different Whole Cereal Flours. Eur. Food Res. Technol..

